# AlphaFold as a prior: experimental structure determination conditioned on a pretrained neural network

**DOI:** 10.1038/s41592-026-03047-4

**Published:** 2026-04-01

**Authors:** Alisia Fadini, Minhuan Li, Airlie J. McCoy, Suresh Banjara, Hiroki Okumura, Eve Napier, Pietro Fontana, Amir R. Khan, Luca Jovine, Thomas C. Terwilliger, Randy J. Read, Doeke R. Hekstra, Mohammed AlQuraishi

**Affiliations:** 1 Cambridge Institute for Medical Research, University of Cambridge, Cambridge, UK; 2 Department of Systems Biology, Columbia University, New York, NY USA; 3 John A. Paulson School of Engineering and Applied Sciences, Harvard University, Cambridge, MA USA; 4 Center for Computational Mathematics, Flatiron Institute, New York, NY USA; 5 Department of Chemistry, Umeå University, Umea, Sweden; 6 Department of Applied Biological Chemistry, Faculty of Agriculture, Meijo University, Nagoya, Japan; 7 School of Biochemistry and Immunology, Trinity College Dublin, Dublin, Ireland; 8 Department of Immunology, St. Jude Children’s Research Hospital, Memphis, TN USA; 9 Department of Medicine, Huddinge, Karolinska Institutet, Stockholm, Sweden; 10 New Mexico Consortium, Los Alamos, NM USA; 11 Department of Molecular and Cellular Biology, Harvard University, Cambridge, MA USA

**Keywords:** Protein structure predictions, Biophysics

## Abstract

Advances in machine learning have transformed structural biology, enabling swift and accurate prediction of protein structure from sequence. However, key challenges persist in modeling side-chain packing, condition-dependent conformational changes and biomolecular interactions, largely because of limited high-quality training data. At the same time, emerging experimental techniques such as cryo-electron microscopy (cryo-EM), cryo-electron tomography (cryo-ET) and high-throughput crystallography are generating vast amounts of structural information but converting these data into mechanistically interpretable atomic models often remains difficult. Here we show that integrating experimental measurements directly into protein structure prediction can overcome these limitations. We introduce ROCKET, an augmentation of AlphaFold2 that refines predicted structures using cryo-EM, cryo-ET and X-ray crystallography data. By optimizing structures in the space of coevolutionary embeddings rather than Cartesian coordinates, ROCKET captures biologically meaningful structural variation that is inaccessible to AlphaFold2 alone and to existing automated modeling approaches, especially when the signal-to-noise ratio is low. ROCKET enables scalable, automated model building without retraining and provides a general framework for integrating experimental observables with biomolecular machine learning.

## Abstract (web summary)

ROCKET improves experimental structure elucidation by integrating implicit structural knowledge from OpenFold, a trainable reimplementation of AlphaFold2, with X-ray crystallographic and cryo-electron microscopy experiments.

## Main

Machine learning (ML) has revolutionized structural biology by enabling highly accurate protein structure prediction. Breakthrough models such as AlphaFold2 (AF2)^1^, RoseTTAFold^2^ and their descendants harness coevolutionary signals in large-scale sequence data to produce predictions with atomic-level precision and near-experimental accuracy^3,4^. Despite this accomplishment, computational approaches still struggle to capture important properties such as side-chain packing, functional dynamics and large-scale molecular assembly^5–9^.

The success of these ML models relies heavily on a vast collection of experimentally resolved structures, made possible by decades of effort by the structural biology community. However, high-quality ground-truth data that capture multiple functional states, biomolecular interactions and structural variations are scarce. Advances in high-throughput experimental techniques are beginning to provide such data. For example, modern crystallography beamlines and single-particle cryo-electron microscopy (cryo-EM) can now yield datasets under many perturbations, for example, for drug screening and tracking of structural changes during biochemical transformations^10,11^, while cryo-electron tomography (cryo-ET) enables in situ observations of macromolecular complexes^12,13^. These advances promise deeper insights into conformational flexibility and complex assembly.

A key bottleneck in processing these large datasets is the reconstruction of atomic models from experimental observations. ML methods have helped streamline this process by providing high-quality starting points for atomic model building^6,14,15^. Standard refinement software^16–18^ improves these starting points by optimizing experimental likelihoods in Cartesian coordinate space, complemented by pattern-matching-based model building to overcome local barriers^19–21^. This combination struggles when the structural rearrangements presented by the data are large, such as switches in secondary structure, flips in flexible loops or shifts in relative domain orientations. Model building becomes particularly challenging, even for humans, below 4–5-Å resolution, where critical structural details such as side chains become indistinct. This difficulty is especially pronounced in the emerging field of cryo-ET, which currently tends to produce data at low resolution^22^. New priors that guide model building could, therefore, yield more accurate atomic models for low-resolution datasets and limit labor-intensive manual intervention when initial structures deviate from the experimental data.

We hypothesized that the implicit prior structural knowledge embedded in pretrained ML structure prediction methods could guide atomic model building from experimental data more efficiently than traditional geometric restraints^23^. Historically, the integration of model and experiment was facilitated by the explicitly physics-based nature of structure prediction methods, which readily permitted the incorporation of experiment-derived potential functions^24,25^. ‘Black-box’ ML models such as AF2, however, make this integration less straightforward. Recent efforts to adapt ML-based structure prediction to incorporate experimental constraints have done so either through fine-tuning of weights from existing architectures^26,27^ or full training of a novel architecture, as exemplified by the ModelAngelo method for cryo-EM data^28^. These strategies are promising but computationally expensive, data hungry and limited to the data modality they were trained on. Another approach, PredictAndBuild^6,29^, iterates between predicting structure conditioned on a template and rebuilding the predicted structures on the basis of experimental data to yield the next template. This approach avoids modifying the structure prediction method but its decoupled prediction and rebuilding steps can work against each other and hinder convergence.

**Fig. 1:**
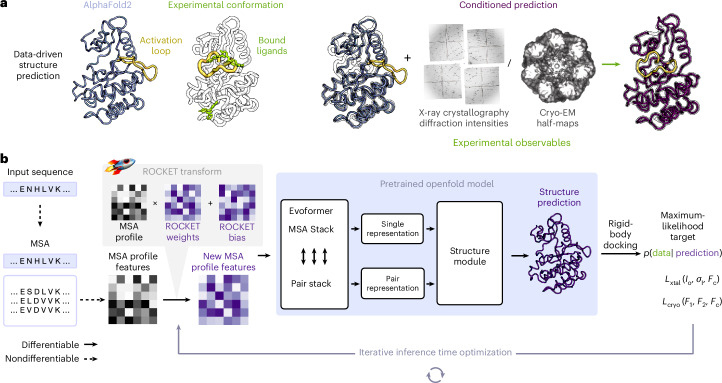
Data-driven structure prediction refinement with ROCKET. **a**, Experimental techniques such as X-ray crystallography and cryo-EM provide high-fidelity observations of conformational states, capturing, for example, ligand-induced changes (right; activation loop of human c-Abl kinase trapped in the inactive state by a small-molecule binder; PDB 3PYY) that may not be modeled by ML-based predictions (left; AF2 prediction of c-Abl in the untrapped state). **b**, ROCKET extends OpenFold by integrating crystallographic and cryo-EM likelihood targets within its differentiable prediction pipeline. It accomplishes this by learning, at inference time, multiplicative and additive adjustments to MSA cluster profiles that maximize their agreement with experimental data as computed by an experimental likelihood function. For the crystallographic target, the function (*L*_xtal_) depends on observed crystallographic diffraction intensities and their measurement errors (*I*_o_, *σ*_I_) and on the structure factor amplitudes computed from the predicted model (*F*_c_). For the cryo-EM target, the function (*L*_cryo_) depends on the complex Fourier terms from experimental half-maps (**F**_1_ and **F**_2_) and the complex structure factors computed from the predicted model (**F**_c_).

Ideally, existing ML methods could serve as implicit structural priors, without retraining, to accelerate and automate atomic model building. Indeed, the ColabDock framework incorporates crosslinking experimental restraints in protein complex structure prediction^30^ and other contemporaneous studies have begun exploring this direction for cryo-EM and crystallography but their applicability to challenging modeling tasks remains unclear^31,32^. Here we combine the high accuracy of pretrained AF2 structure prediction with guidance from experimental data in a way that can be flexibly adapted to different data modalities. We achieve this through ROCKET (refining OpenFold with crystallographic/cryo-EM likelihood targets), a framework that integrates OpenFold^33^, a trainable reimplementation of AF2, with differentiable crystallographic and cryo-EM likelihood targets^34^. ROCKET refines structures at inference time (no retraining), steering predictions toward experimentally supported conformations (Fig. 1a). Inspired by strategies for expanding AF2 to conformational sampling^35–39^, we directly optimize the embedded multiple-sequence alignment (MSA) cluster profile^36,37^ in AF2, transforming structure refinement to a data-guided search within AF2’s continuous representation of sequences.

Cryo-EM and crystallography are the predominant techniques for producing atomic-level insights into conformational heterogeneity, macromolecular interactions and functional structural rearrangements. Despite crystallography’s promise for high-resolution, high-throughput screening of experimental conditions^40–42^ and drug candidates^43^, ML applications in protein crystallography^34,44^ lag behind cryo-EM^28,45–47^. To bridge this gap, ROCKET integrates both X-ray and cryo-EM data directly into OpenFold’s inference process.

For both data modalities, we find that ROCKET is particularly valuable for the unsolved challenge of model building at low resolution^48^. Existing software struggles at resolutions worse than 4–5 Å; ModelAngelo was not trained to work with maps past 4 Å (ref. ^28^) and PredictAndBuild will not automatically rebuild with maps worse than 3.5 Å (ref. ^6^). We find that ROCKET’s inference-time refinement allows it to explore a wide conformational space and it remains robust for atomic modeling in noisy, low-resolution maps, sometimes outperforming expert manual refinement. As ROCKET relies on an input sequence for AF2 inference, it is complementary to other ML approaches designed to identify proteins in cryo-EM maps^28,49,50^. Its capabilities render ROCKET a generalizable approach for integrating experimental data with ML-based biomolecular modeling.

## Results

### Method overview

AF2-based structure prediction begins with the construction of an MSA, typically through a search of sequence databases for proteins homologous to the query of interest. The resulting MSA comprises aligned sequences of identified homologs, from which one can infer a protein’s evolutionary history, including its patterns of residue–residue coevolution. AF2 transforms raw MSAs into an input representation suitable for neural network computation, known as the MSA cluster profile. Through many studies and ablations, it has been shown that the depth and diversity of the MSA and the statistical patterns found therein determine the geometry and quality of a structure predicted by AF2 (ref. ^1^). Given this central role, we reasoned that direct optimization of the continuous space of MSA cluster profiles would provide the greatest lever for influencing AF2 predictions, a hypothesis supported by previous work on using evolutionary coupling restraints to build molecular replacement templates^51^, recent observations made by other groups^36,37^ and our own experiments (Supplementary Fig. 1).

To operationalize this principle, ROCKET augments OpenFold with a new module that optimizes MSA cluster profiles to maximize agreement between predictions and experimental observables (Fig. 1b and Methods). Through an experimental target function that quantifies this agreement, ROCKET performs gradient descent in the space of MSA cluster profiles. During each descent step, ROCKET computes a forward pass through OpenFold, evaluates the target function and its derivatives and then updates the MSA cluster profile to increase the function’s agreement with model predictions. ROCKET currently provides two target functions, for crystallographic and cryo-EM data, and is extensible to other data modalities.

As OpenFold-predicted structures are generated in an arbitrary reference frame, ROCKET first performs molecular replacement^52^ or cryo-EM docking^53^ to align the predicted model with the experimental data before starting the iterative refinement process. The resulting rototranslation is applied at every subsequent iteration to align the model. While initial AF2 predictions are less than perfect, previous observations indicate that they are usually sufficiently accurate for robust placement in the data^14,15,29,54^. After iterative refinement through OpenFold is complete, a final local structure refinement is performed using phenix.refine^16^ to optimize local geometry and atomic displacement parameters. We investigate the impact of ablations of the ROCKET pipeline in Supplementary Fig. 5. Because of memory limitations, ROCKET currently only operates on one protein chain at a time and requires special handling for cases involving multiple chains (Methods).

### Evaluation dataset and approach

We took a two-pronged approach to evaluating ROCKET’s effectiveness in guiding structure prediction with experimental data. In the first prong, we validated ROCKET’s model-building accuracy across a range of resolutions. We started by confirming that ROCKET could match the best established methods for high-resolution X-ray datasets. For such cases, ROCKET’s primary utility would be in streamlining experimental model building through integration with ML-based structure prediction. We then generated two reduced-resolution cryo-EM series—by either omitting or degrading experimental data—that allowed us to assess and develop confidence in ROCKET’s model building at progressively lower resolutions. In the second and more ambitious prong, we turned to scenarios at the frontier of experimental structural biology, where automated methods typically fail, cases in which manual human intervention is necessary and may not even be sufficient.

For the first prong, we started by identifying a diverse set of 27 high-resolution, single-chain X-ray crystallographic datasets and their corresponding deposited structures (Supplementary Fig. 2). All 27 structures were solved after the AF2 training date cutoff and at resolutions better than 3 Å (Supplementary Table 1), a regime where conventional methods, such as phenix.refine, perform well and hybrid methods, such as PredictAndBuild, perform extremely well^29^. To then study ROCKET’s performance when large-scale structural changes are required from the starting AF2 prediction, we selected ligand-induced loop rearrangements in three human proteins: c-Abl kinase (PDB 3PYY), protein tyrosine phosphatase 1B (PDB 1NWL) and the serpin plasminogen activator inhibitor-1 (PDB 1JL5). At high resolution, we prioritized crystallography because, as noted earlier, its integration with ML-based methods remains underdeveloped^34^.

Anticipating that, for cryo-EM, ROCKET’s added value would manifest at lower resolutions, we constructed reduced-resolution series where the accuracy of ROCKET models built from progressively weaker experimental signals could be objectively evaluated against a higher-resolution map that serves as the ground truth. As a first example, we chose the thiamine transporter SLC19A3 structure solved from a single-particle 3.1-Å map in the outward-open state (PDB 8S4U, EMD-19716)^55^. We added noise separately to each high-resolution half-map until their Fourier shell correlation (FSC) curve matched that expected of lower-resolution maps at 6.0 Å, 8.0 Å and 10 Å (Methods). As a second example, we picked a time-resolved single-particle 20-s intermediate of the GroEL:GroES–ATP complex, solved at 2.7 Å (PDB 8BM1, EMD-16117)^56^. We reprocessed progressively smaller subsets of the particle images to produce maps at resolutions of 2.9 Å, 4.9 Å and 6.8 Å. For these two series, we then tested how well ROCKET (which, in both cases, starts from an AF2 prediction that is substantially different in conformation) recovers the experimentally captured state from maps progressively decreasing in quality.

For the second prong, we tackled the following challenging model-building case studies: a 9.60-Å subtomogram average of *Escherichia* *coli* GroEL (PDB 8P4P), a 3.82-Å, crystallographic dataset of the human multidomain protease inhibitor HAI1 (PDB 5H7V), a 3.5-Å cryo-EM dataset of 2:2 complex of human protein phosphatase PPM1H and its substrate Rab8a, complicated by preferred particle orientation, and a time-resolved extrapolated dataset of class II photolyase bound to a thymine dimer, captured 10 μs after initiation of DNA repair (PDB 8OYA). Lastly, we highlight a case where ROCKET provided biological insight by supporting multimeric model building into a single-particle 8.6-Å map and where the resulting ROCKET structure was later validated against a higher-resolution 4.6-Å dataset.

### ROCKET reliably models structural details across a wide resolution range

**Fig. 2:**
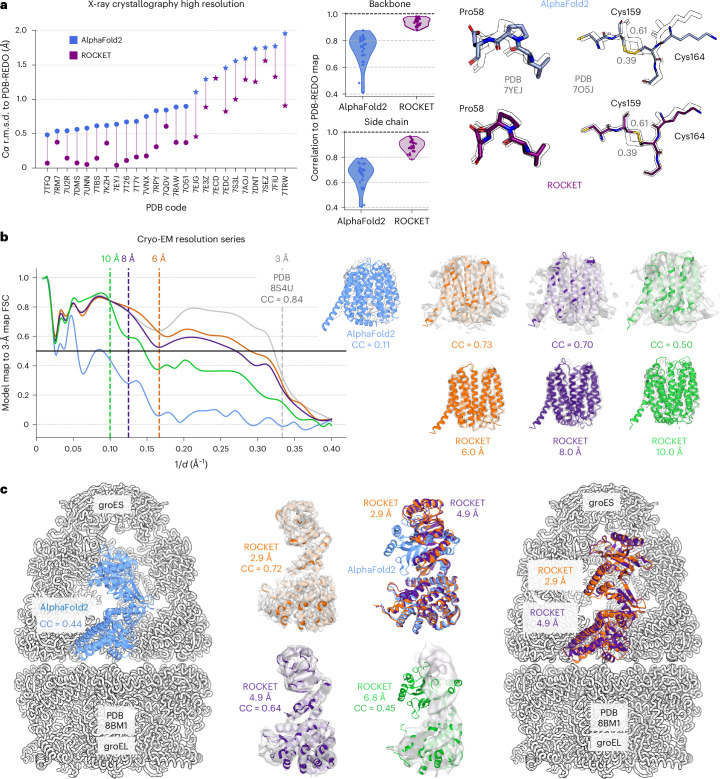
ROCKET reliably models structural details across a wide resolution range. **a**, High-resolution crystallographic set (27 structures): Cα r.m.s.d. of AF2 versus ROCKET relative to PDB-REDO; ROCKET reduces r.m.s.d. in all but one case (stars mark the ten most difficult; mean drop of 0.47 Å). RSCC for backbone and side chains shows systematic improvement after ROCKET. Right, two examples highlight ROCKET’s ability to sample changes that overcome structural barriers. A peptide flip in the refinement of *E*. *coli* nucleoside phosphorylase (PDB 7YEJ). A switch in a disulfide bond in the structure of thaumatin from *Thaumatococcus* *daniellii* (PDB 7AOJ). Two alternate conformations are present in the deposited structure, with refined occupancies of 0.39 and 0.61. ROCKET builds the conformation with the highest occupancy. **b**, SLC19A3 thiamine transporter at 6, 8 and 10 Å: ROCKET converts the inward-open AF2 model to the outward-open state and improves RSCC for all three resolutions. The FSC between refined-model maps and an independent 2.9-Å map remains >0.5 to 3.56 Å (6 Å) and 3.66 Å (8 Å); at 10 Å, increased rigid-body positioning ambiguity reduces agreement, yet FSC stays >0.5 to 7.14 Å. **c**, Time-resolved GroEL:GroES–ATP series; refining the GroEL subunit requires a large top-domain rearrangement. ROCKET recovers the complexed conformation at 2.9 Å and 4.9 Å (RSCC to the deposited 2.7-Å map improves from 0.44 to 0.72 and 0.64, respectively). At 6.8 Å, lower-domain placement improves but the full rearrangement is not recovered, consistent with gradient-descent trapping in a local minimum (if sampled, the correct conformation would achieve a higher experimental likelihood; LLG = 1,583 versus LLG = 1,448 for the ROCKET model).

At high resolution, we expect deposited backbone and side-chain coordinates to serve as reliable ground truths for evaluation. To validate ROCKET’s performance on the 27 crystallographic structures in our first prong, we compared its refined models to those from the PDB-REDO database^57^, which systematically rerefines and validates human-deposited coordinates. Figure 2a shows the Cα root-mean-squared deviation (r.m.s.d.) with respect to PDB-REDO models for original AF2 predictions and ROCKET-refined ones. ROCKET improves all AF2 predictions except one, bringing them closer to the experimentally determined structures. Focusing on the ten most difficult cases, where AF2 predictions deviated by more than 1 Å r.m.s.d. from the PDB-REDO models, ROCKET achieves substantial structural corrections (Fig. 2a, stars; mean r.m.s.d. drop of 0.47 Å), demonstrating robustness in challenging scenarios.

Although Cα r.m.s.d. is a convenient measure of overall model quality, it heavily penalizes complete models that retain less ordered regions when compared to reference models that omit those regions. For a more sensitive assessment, we used the real-space Pearson correlation coefficient (RSCC), which directly compares model-derived electron density maps to maps from experimental amplitudes (here, combined with phases from PDB-REDO models). As shown in Fig. 2a (middle), ROCKET substantially improves RSCC values for both backbones and side chains across all test cases. It is notable that optimizing MSA cluster profiles not only corrects secondary structures but also improves side-chain fit to the data (Supplementary Fig. 3). On this benchmark, ROCKET achieves RSCC accuracy comparable to human-deposited models and performs on par with PredictAndBuild and with ModelCraft, an advanced de novo automated model-building pipeline for crystallography and cryo-EM datasets^58^ (Supplementary Fig. 4a). In further validation using crystallographic *R*_free_ factors^59^ (Supplementary Fig. 4b), all but two models see improvements relative to phenix.refine alone, with ten models showing an *R*_free_ reduction of more than 3%. We note that ROCKET achieves performance on *R*_free_ comparable to both ModelCraft and to the more complex combination of PredictAndBuild + phenix.refine, while still maintaining, unlike the other two methods, full model sequence completeness (Supplementary Fig. 4c).

We found that performing optimization in the latent MSA profile embedding allows for more pronounced structural rearrangements than conventional refinement in Cartesian coordinate space; compared to phenix.refine, ROCKET is able to better recover experimental fold and backbone from AF2 predictions with larger initial r.m.s.d. to the deposited structure (Supplementary Fig. 5). For the high-resolution benchmark, internal data-driven iterative updates to AF2’s prediction (ROCKET) are comparable to external rebuilding iterations (PredictAndBuild) (Supplementary Fig. 6a). ROCKET, however, can perform challenging large-scale structural rearrangements, such as ligand-induced loop movements and peptide flips, that are not accessible to PredictAndBuild (Supplementary Fig. 6b–e). We highlight that these two strategies can complement each other (Supplementary Fig. 6f). Additionally, in cases where the initial prediction requires a large structural change, subsampling MSAs to generate alternative MSA profile embeddings can improve initial predictions and help overcome limitations of gradient descent (Supplementary Figs. 7 and 8).

After benchmarking ROCKET at high resolution, we evaluated its model-rebuilding performance at progressively lower resolutions. For the SLC19A3 thiamine transporter series, ROCKET recovers the outward-open conformation from an inward-open AF2 starting model even at 10 Å. The overall fold is recovered at all three target resolutions (6 Å, 8 Å and 10 Å), and is supported by the largely improved RSCC values for the final models (Fig. 2b). After full ROCKET refinement, we computed FSC curves between maps calculated from the refined models and the independent, original 2.9-Å map that was not used for refinement. In each case, the refined models agree with the high-resolution data well beyond the nominal resolution of the target maps; models refined against 6-Å and 8-Å data maintain FSC > 0.5 up to 3.56 Å and 3.66 Å, respectively. At 10 Å, ambiguity in rigid-body placement within the map reduces agreement relative to the other two cases. Nevertheless, the 10-Å model retains FSC > 0.5 up to 7.14 Å. These results indicate that ROCKET can capture high-resolution detail absent from the guiding maps, showing that the AF2 structural prior provides information complementary to the experimental signal.

We next examine the time-resolved GroEL:GroES–ATP series, whose results are summarized in Fig. 2c. We refine the GroEL subunit from the upper heptameric ring, where, compared to the starting AF2 prediction, the top domain needs to undergo a large rearrangement to complex with the respective GroES subunit. At 2.9 Å and 4.9 Å, ROCKET recovers the complexed conformation, improving RSCC against the deposited 2.7-Å map from 0.44 (AF2) to 0.72 (2.9-Å-refined ROCKET) and 0.64 (4.9-Å-refined ROCKET). This task is more challenging than the thiamine transporter above because the required torsion of the top domain weakens the gradient-based signal. The limitation becomes evident at 6.8 Å, where ROCKET improves placement of the lower domain but does not recover the full conformational change. Although the correct conformation would achieve a higher experimental log-likelihood gain (LLG; 1,583 versus 1,448 for the ROCKET model), gradient-descent refinement remains trapped in a local minimum.

### Data-driven MSA profile optimization improves AF model confidence

Intriguingly, we noticed that, across our crystallographic datasets, ROCKET’s AF2-derived model confidence (measured as per-residue predicted local distance difference test (pLDDT)) is correlated with agreement with experimental data, as reflected in the positive correlation between final model confidence and RSCC (Supplementary Fig. 9). As MSAs may implicitly encode multiple conformations that a protein can adopt^35^, we suggest that ROCKET’s data-guided optimization uses and then resolves this structural ambiguity, allowing it to reach different functional conformations. To more directly test this hypothesis, we performed a (negative) control experiment. AF2 includes built-in confidence metrics, which several studies have leveraged to explore conformational space, particularly in the context of protein design^36,60–62^; we asked whether optimizing MSA cluster profiles with respect to AF2’s own confidence metrics may improve structure prediction without the need for experimental data. To implement this idea, we used ROCKET to optimize pLDDT, AF2’s primary confidence metric. We found that this approach fails to improve the correspondence of AF2 predictions with experiment in every one of the cases studied (Supplementary Fig. 10), indicating that experimental data provide new and orthogonal information, beyond AF2’s implicit scoring function, which is necessary for efficient sampling of functionally relevant conformations. This clarifies but does not contradict our first finding. Specifically, it indicates that pLDDT can identify highly preferred conformations but cannot, by itself, distinguish the experimentally observed state (and provide a useful gradient) without further experimental information.

### ROCKET facilitates frontier model-building tasks

**Fig. 3:**
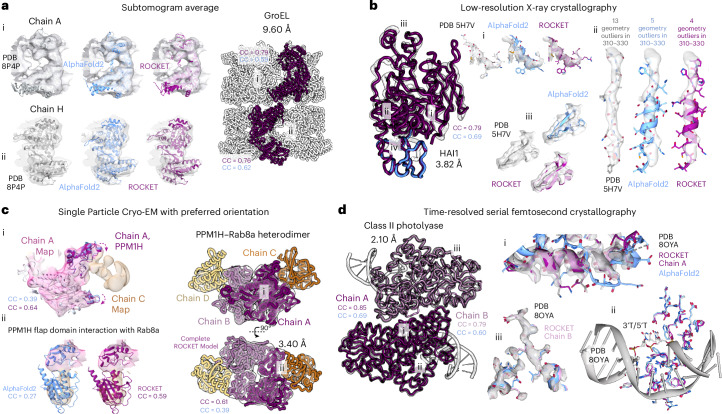
ROCKET facilitates frontier model-building tasks. For four challenging model-building examples, AF2 predictions (blue), deposited models (gray) and ROCKET-refined models (purple) are shown. Mean RSCC values to the experimental map are reported before and after ROCKET rebuilding. **a**, *E*. *coli* GroEL subtomogram average at 9.60 Å. Two conformations of the repeating subunit can be distinguished in the top and bottom heptameric rings. The per-residue RSCC is plotted in Supplementary Fig. 11. **b**, Human HAI1 crystallographic dataset at 3.82 Å. For regions i–iii, AF2, deposited and ROCKET-refined backbones are compared through local density fit to the PDB-REDO map; region iv, which is poorly resolved and not modeled in the deposited structure, is left unchanged by ROCKET. **c**, PPM1H–Rab8a dimeric complex from a 3.4-Å cryo-EM map affected by preferred orientation. A single PPM1H (chain A) and Rab8a (chain C) were built independently before applying symmetry operations to generate the full 2:2 complex. The figure highlights key improvements over AF2 predictions, demonstrating that ROCKET (i) captured a literature-supported conformational change in the PPM1H active site and (ii) substantially improved the modeling of the flap domain’s interaction with Rab8a, as measured by RSCC. Further comparisons with other modeling methods on this task are shown in Supplementary Fig. 12. **d**, Time-resolved extrapolated dataset for class II photolyase DNA (PDB 8OYA), collected 10 μs after initiation (nominal resolution of 2.10 Å, effective resolution of ~2.5–2.8 Å). Two independent complexes (chains A and B) are refined by ROCKET; examples are shown for helix 200–214 (i) and the DNA-binding interface (ii). Refinement used no explicit DNA information. (iii) ROCKET rebuilds a solvent-exposed segment of chain B where the density is markedly poorer than in chain A, recovering side-chain rotamers and a backbone shift that, where supported by density, match the depositing group’s choices.

Cutting-edge structural biology techniques often generate data at the limits of available methodology, with partially automated workflows bottlenecked by human expertise. Examples include (1) cryo-ET, where missing-wedge artifacts and noise obscure atomic detail; (2) low-resolution crystallography, where conformational heterogeneity from flexible loops and domains blurs density and increases susceptibility to phase bias during refinement; (3) cryo-EM with a preferred orientation of particles, which results in an incomplete sampling of views that compromises the final three-dimensional (3D) reconstruction; or (4) the emerging field of time-resolved crystallography, where signals from short-lived intermediates must be disentangled from mixtures of activated and ground-state populations^63^. To assess whether ROCKET can address these frontier problems, which remain solidly outside the purview of existing methods, we tackled four representative tasks summarized in Fig. 3.

We begin with two datasets that lie past the resolution limits for automated model building: a 9.60-Å subtomogram average of *E*. *coli* GroEL and a 3.82-Å human HAI1 crystallographic dataset, where domain motion prevented high-resolution diffraction (Fig. 3a,b). In the GroEL subtomogram average, the 9.60-Å map reveals two distinct conformations for the repeating subunit in the top and bottom heptameric rings. Despite the low resolution, ROCKET accurately recovers both conformations (Fig. 3a), yielding refined models with map correlations comparable to deposited structures (Supplementary Fig. 11). Moreover, for chain A, ROCKET explores a broad conformational space, deviating substantially from the AF2 prediction. Notably, in the top domain (residues 250–280; star in Supplementary Fig. 11), ROCKET achieves an average RSCC of 0.5, substantially higher than the 0.0 achieved by the AF2 model or even the 0.2 achieved by the deposited model, supporting a closer match to experimental data.

In well-resolved regions of HAI1, ROCKET successfully converges to a backbone conformation that more closely matches the experimental density than the AF2 prediction (Fig. 3b, regions i–iii). Particularly noteworthy is region iii (residues 310–330), where the density is highly noisy, making even manual model building difficult. In this segment, the deposited model appears to have an incorrect sequence register. ROCKET corrects this and improves AF2’s initial prediction to better align with the density map without introducing new geometric outliers. Conversely, in areas where the experimental map is poorly resolved, such as region iv, which is not modeled in the deposited structure, ROCKET refrains from forcing arbitrary changes and instead preserves the original AF2 prediction.

Next, we modeled the 2:2 heterotetrameric complex of the protein phosphatase PPM1H and its substrate Rab8a (Fig. 3c) using ROCKET from a 3.5-Å cryo-EM map affected by preferred orientation (Supplementary Fig. 12). This dimeric assembly represents the physiological state of the enzyme, which creates a more intricate interaction surface than a monomer can provide^64^. AF3 (ref. ^9^) fails to produce a plausible complex (Supplementary Fig. 12), underscoring the need for map-guided modeling to reveal the correct physiological assembly. Compared to a docked AF2 prediction, the ROCKET model achieves a much higher real-space correlation coefficient (CC) with the cryo-EM map (Supplementary Fig. 12). Specifically, ROCKET captures a crucial conformational change in PPM1H, where the highly conserved flap domain (region i, top part, residues 304–414) moves toward Rab8a. This movement induces a change in the active site consistent with previously reported flexibility^65^. Furthermore, ROCKET substantially improves the modeling of the flap domain–Rab8a interface (region ii), which is the primary determinant of substrate specificity^64^. ROCKET also positions the β-motif (region i, bottom part) distant from Rab8a, suggesting that it is not directly involved in this interaction; both findings are consistent with biochemical evidence (Supplementary Fig. 13). We benchmarked our result against the de novo modeling tool ModelAngelo^28^, as this 3.4-Å dataset falls within its applicable resolution range. Although ModelAngelo improved the model–map CC in the regions it built, its model suffered from low completeness (<20%). In contrast, ROCKET achieved the highest overall CC value while providing a complete model of the complex.

For our final frontier task, we used ROCKET to refine a time-resolved extrapolated dataset of class II photolyase bound to a thymine dimer (Fig. 3d), collected 10 μs after initiation of DNA repair (PDB 8OYA). Although the dataset has a nominal resolution of 2.10 Å, the effective resolution of the extrapolated data is estimated around 2.50–2.80 Å (Supplementary Table 1)^66^. The crystals contain two independent protein–DNA complexes; chain A exhibits stronger time-resolved signal and consistently lower *B* factors (by 10–20 Å^2^ in some regions) than chain B, likely because of crystal packing. We report ROCKET refinements for both chain A and the more challenging chain B. In both cases, ROCKET markedly improves model–map agreement (Fig. 3d). Per-residue RSCC values comparing AF2, ROCKET and deposited structures are plotted in Supplementary Fig. 14. In the helical segment spanning residues 200–214, ROCKET correctly rebuilds the secondary-structure element (region i), with remaining differences relative to the deposited model confined to solvent-exposed side chains. At the DNA-binding interface, ROCKET robustly corrects side-chain-level details (region ii), guiding AF2 toward the DNA-bound conformation. This happens without providing any explicit information about DNA during inference and is purely driven by protein-specific data. ROCKET also rebuilds a solvent-exposed segment of chain B where the density is markedly poorer than in chain A (Fig. 3d, region iii), recovering side-chain rotamers and a backbone shift that, where supported by density, match the depositing group’s choices. These results collectively highlight ROCKET’s utility for structural interpretation from heterogeneous or weak signals.

### ROCKET accurately models the structure of a homomeric protein filament

Beyond the four frontier challenges we just described, we sought to apply ROCKET to an active project involving multimeric model building from a low-resolution cryo-EM map. Subsequent to our model building, we obtained a higher-resolution dataset that validated ROCKET’s structure.

Vertebrate reproduction depends on the zona pellucida (ZP), a specialized extracellular coat that surrounds the egg and, from amphibians to mammals, mediates sperm attachment, penetration and the block to polyspermy^67^. The ZP is a 3D mesh of filaments assembled by glycoprotein subunits that share a ZP module, a polymerization element consisting of ZP-N and ZP-C domains separated by an interdomain linker (IDL)^68^. Recently, the structure of a heteromeric egg coat filament was obtained from native ZP fragments that crystallized by reassembling into polymers^69^. However, the paucity, highly heterogenous nature and often covalent crosslinking of ZP filaments has made it challenging to obtain detailed structural information for intact filaments.

**Fig. 4:**
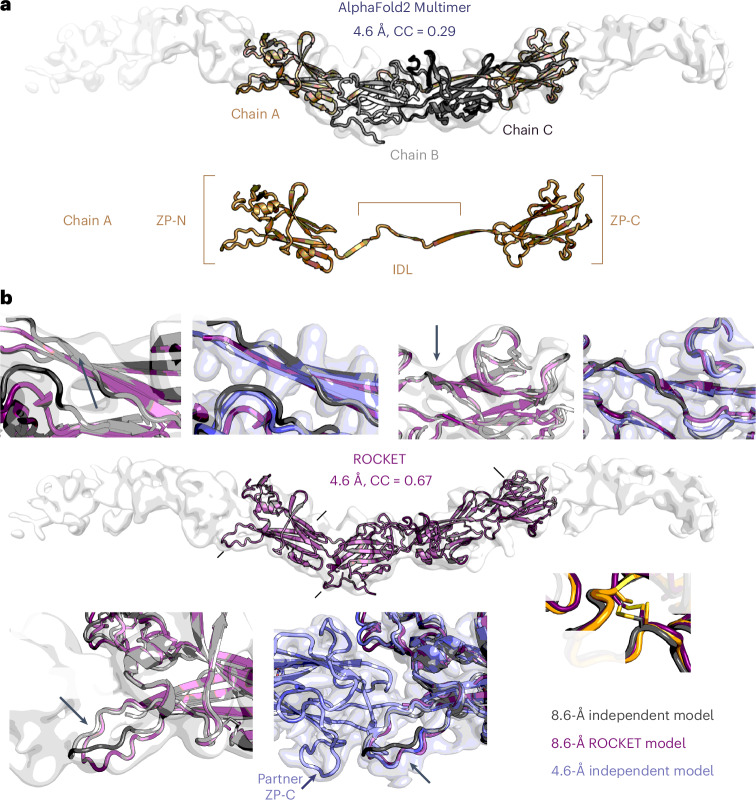
ROCKET enables model building of a ZPD filament from low-resolution cryo-EM. **a**, An AF2 trimer docked into an 8.6-Å helical reconstruction of avian ZPD; for visualization purposes only, the map is shown postprocessed with EMReady2 (ref. ^71^). The ZP module is a polymerization element consisting of ZP-N and ZP-C domains separated by an IDL. Here, a central subunit (chain A; ZP-N–IDL–ZP-C) engages the ZP-C of the preceding subunit (chain B) and the ZP-N of the following subunit (chain C), matching the intersubunit register observed in vertebrate ZP polymers^69^. **b**, Comparison of the ROCKET-built model (purple), refined solely against the 8.6-Å unsharpened half-map data, with (i) an independently built model without ROCKET, based on the same low-resolution map (Methods), and (ii) a higher-resolution 4.6-Å single-particle analysis ZPD map and corresponding model (light blue), both produced without ROCKET. The ROCKET model shows improved global agreement to the 4.6-Å map (CC = 0.67) relative to the unrefined AF2 prediction (CC = 0.29) and recovers key architectural features validated by the higher-resolution data: (i) a more accurately positioned ZP-N fg loop interacting with a complementary surface of the partner ZP-C, (ii) IDL conformational details that stabilize intersubunit contacts, and (iii) better-defined secondary structure in regions that were ambiguous at 8.6 Å. (iv) A disulfide that appeared broken in the unrelaxed ROCKET prediction but was correctly resolved by a polishing step with conventional refinement.

We used ROCKET to aid model building into a 8.6-Å helical reconstruction map of ZPD^70^, a noncrosslinked, homopolymeric glycoprotein of the avian egg coat. In Fig. 4a, we illustrate the docked AF2 prediction, in which a ZP module (chain A) engages with the ZP-C and ZP-N domains of the preceding and following subunits within the filament, respectively (chains B and C), as observed in the fish ZP heteropolymer^69^. For visual clarity, we display a map postprocessed using EMReady2 (ref. ^71^), although we emphasize that ROCKET refinement was carried out against unmodified half-map data. In Fig. 4b, we compare the ROCKET-built model (purple) to a model whose initial coordinates also came from AF2 but that was independently built without ROCKET on the basis of the same low-resolution map (Methods). Importantly, our validation can also take advantage of a higher-resolution 4.6-Å map of ZPD obtained by single-particle analysis of a later dataset and a corresponding model also refined without ROCKET (light blue model and map). ROCKET’s model, refined solely against the 8.6-Å map, achieves a CC of 0.67 to the higher-resolution map compared to only 0.29 for the unrefined AF2 prediction. Beyond this overall agreement, ROCKET captures key architectural features that differ from the independently built 8.6-Å model but are validated by the higher-resolution dataset. These include (1) a much more accurately modeled ZP-N fg loop interacting with a complementary surface of the partner ZP-C; (2) conformational details in the IDL that stabilize subunit interactions within the filament; and (3) better-defined secondary structure in regions where the low-resolution model was ambiguous. Underscoring the importance of the full pipeline and consistent with the ablation study in Supplementary Fig. 5f, we show a broken disulfide from the unrelaxed ROCKET model prediction, which was resolved by a conventional refinement polishing step (inset iv).

This validation against independently collected higher-resolution data provides a strong demonstration of ROCKET’s ability to recover accurate atomic information, even under the challenging conditions of low-resolution cryo-EM. Biologically, the structure of native ZPD provides important evidence that all egg coat protein filaments share a common general organization, with the same interlocked subunit architecture first observed for urinary ZP module protein uromodulin^72^ but with helical twist parameters around −120° (ref. ^69^).

## Discussion

In developing ROCKET, we demonstrate that experimentally guided refinement of the embedded MSA profiles of AF2 enables efficient exploration of conformational space. Our results suggest that these embeddings provide access to paths along which the barrier for structural rearrangements is greatly reduced or eliminated, indicating that information about such rearrangements may be encoded in evolutionary statistics. Furthermore, we show examples where leveraging AF2 priors in combination with experimental constraints extends atomic model building into increasingly complex and dynamic structural datasets.

We anticipate that data-guided, inference-time optimization will prove broadly valuable across diverse atomic model-building scenarios. Beyond cryo-EM and crystallographic datasets, implemented here, our approach can be used for other, sparser data modalities, provided a likelihood-based target between data and prediction can be formulated. Extending ROCKET to handle multichain complexes or protein–ligand cofolding is straightforward within any AF-like framework, in principle requiring only a switch in the inference model used.

More ambitious directions may involve integrating generative models to account for conformational ensembles. Furthermore, given our success with MSA profile biasing, we propose that learning a mapping from experimental observables to a profile bias matrix could effectively condition single-shot structure prediction toward experimentally probed conformations. Such a mapping would translate experimental data, such as cryo-EM density maps, X-ray diffraction intensities or nuclear-magnetic-resonance-derived constraints, into a profile bias matrix that captures residue-level probabilities or pairwise constraints derived from experiment, effectively guiding the model toward relevant conformations without requiring exhaustive searches. By adopting amortized inference, which learns a reusable mapping to enable fast predictions for new inputs, we could further streamline the process by replacing the stepwise search (MSA subsampling and likelihood scoring; Supplementary Fig. 7) with a more efficient learned transformation.

**Fig. 5:**
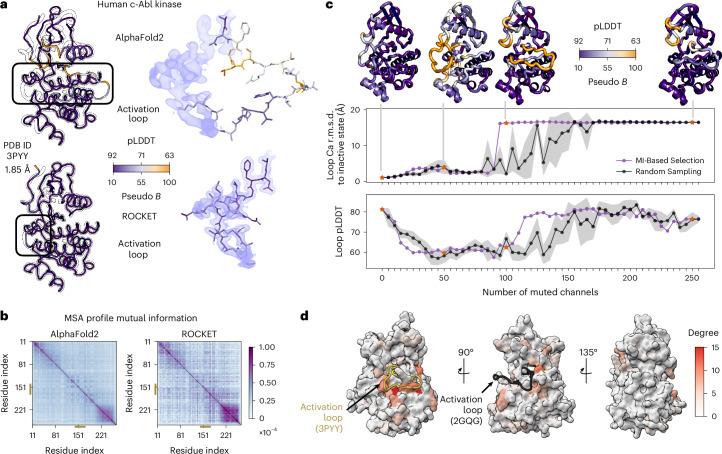
MI highlights distributed residue signals driving conformational transitions. **a**, Structural comparison of human c-Abl kinase in two states. Top, AF2 prediction, representing the active state. Bottom, ROCKET-refined model, consistent with the inactive conformational state (PDB 3PYY, 1.85-Å resolution, shown in transparent outline). Both structures are colored by pLDDT, with P-loop (residues 13–28) and activation loop (residues 148–167) highlighted with corresponding ligand-induced experimental density. **b**, Heat maps of MI matrices derived from MSA profiles, both normalized. Left, MI matrix from the initial AF2 MSA profile. Right, MI matrix from the ROCKET-refined MSA profile. Differences in these matrices highlight how coevolutionary signals are altered during refinement to enable the conformational transition. **c**, Causal testing through profile bias muting. Top, ROCKET model shown at progressive stages of muting. Middle, activation loop Cα r.m.s.d. to the inactive state versus the number of muted channels. Muting channels on the basis of MI difference (purple) reverts the structure to its active state more effectively than random muting (gray; mean and s.d. over five independent trials per point). Bottom, corresponding pLDDT of the activation loop. Reversing the flip requires muting roughly one third of the channels, indicating that the conformational signal is diffuse rather than localized. **d**, MI-guided muting of the top 155 ∥ΔMI∥ residue pairs (95 unique residues) reverted the protein structure. Residues are color-coded by their ‘degree’ (that is, the frequency of their appearance in these top 155 pairs). The involved residues are mostly localized at the bottom interface of the kinase with the ‘inactive’ A-loop (yellow), with some residues participating at the interface of the kinase with the ‘active’ A-loop (black).

To understand how ROCKET alters its coevolutionary representation during optimization, we examined the signals within the refined MSA profile that contribute to a specific structural transition: the prototypical conformational change of the activation loop of c-Abl kinase between its inactive (observed here) and active conformation. ROCKET’s prediction confidence increased notably during refinement (Fig. 5a), consistent with the hypothesis that ROCKET’s optimized MSA profile disambiguates multistate coevolutionary signals into a clear structural directive. To identify the coevolutionary features most impacted by ROCKET’s MSA profile optimization, we computed residue–residue correlations, in the form of mutual information (MI) matrices, from the initial and the final MSA profiles. The correlations in the final MSA profile are more dispersed than in the original (Fig. 5b). To understand the causal role of changes in the MSA profile, we first ranked residue pairs on the basis of the magnitude of change in their correlations implied by the MSA profile (by ∥ΔMI∥) and then, starting from the final ROCKET MSA profile, progressively muted the optimized signals at these high-ranking residue pairs by replacing them with their corresponding values from the untransformed MSA profile. This MI-guided muting strategy proved much more effective at reverting the structure back toward its incorrect starting conformation, inducing a flip with ~95 muted residues, relative to a random sampling baseline that required ~170 residues (Supplementary Fig. 5c). In contrast, muting residues on the basis of their rank by simpler statistics such as the position-specific scoring matrix entropy change or average absolute MI change did not outperform random selection (Supplementary Fig. 15a), indicating that changes to specific pairwise correlations drive ROCKET’s performance. Further analysis of the top-ranked pairs revealed that the signal is not uniformly distributed but is instead concentrated; the top 155 pairs in the ∥ΔMI∥ ranking involve fewer unique residues than 155 randomly selected pairs (Supplementary Fig 15b). As shown in Fig. 5d, the involved residues cluster at the interface ‘below’ the activation loop and at contact points for the active conformation of the loop (superposed from PDB 2GQG), while residues at the ‘back’ of the kinase are hardly affected. Nevertheless, nearly one third of the entire protein is involved in some of the residue–residue correlations that need to be muted to undo the activation loop’s conformational switch, reinforcing the challenge of engineering such transformations manually and motivating the development of learned conditioning approaches.

While additional work is needed to fully realize these extensions, our study marks critical progress by showing that structure optimization in coevolutionary embeddings can overcome limitations of conventional refinement. Additionally, by introducing differentiable likelihood targets for cryo-EM and crystallography, which include a robust treatment of measurement error, we provide a framework that is well suited for training future predictive models. More broadly, our Bayesian approach to combining information learned by ML models with information obtained by direct observation establishes a foundation for continuous interaction between ML and experimental data. This interplay is critical for the interconnected goals of scaling experimental throughput and training ML models with enhanced functionality.

Our atomic model building is automated, requires no retraining and, for difficult cases, produces models of quality comparable or even superior to those created by expert human modelers. Despite this advance, certain limitations remain. First, it is always advisable to inspect refined models visually and to validate critical aspects of the model by complementary data when the experimental map is of low quality. For crystallographic cases, the current approximation of the atomic displacement parameters is derived empirically from model confidence and could be improved by incorporating considerations of density fit at the residue level. Additionally, because OpenFold is not explicitly aware of crystal contacts, ROCKET may struggle to converge to certain lattice-constrained conformations. We expect that this limitation can be addressed by extending ROCKET to use multichain models^73^. We also noticed that ROCKET can fail to flip small loops (3–4 residues in length) that contain bulky side chains. We show examples of cases that are difficult for ROCKET in Supplementary Fig. 16. Perhaps most importantly, iterative backpropagation through OpenFold is memory intensive and limits the maximum size of the protein or domain that can be refined at once—about 500 residues on a 40-GB A100 GPU (Supplementary Fig. 17). We believe that this can be extended with further code optimization and the implementation of a learned mapping for single-shot MSA profile bias, mentioned above, could also help overcome this limit.

Naturally, assessing biological accuracy solely through the lens of how well the atomic model fits experimental maps has inherent limitations, as both cryo-EM and crystallography can introduce artifacts^74^. Ultimately, multimodal information is essential for building a full picture of physiological protein states and functions. For this purpose, our approach is readily adaptable to alternative loss functions that combine multiple sources of experimental and computational data, supporting integrative modeling strategies for biological structure determination.

## Methods

### ROCKET algorithm and processing pipeline

ROCKET’s inputs and preprocessing steps are summarized in Algorithm 1, while the ROCKET inference-time optimization algorithm is summarized in Algorithm 2.

#### Inputs and preprocessing pipeline

For crystallographic datasets, a protein sequence and a reflection MTZ file (or CIF file) containing observed intensities and their uncertainties are required; for cryo-EM datasets, two half-maps are required. As outlined in Algorithm 1, to obtain an aligned reference model (**x**^ref^), we use Phasertng^75^ for molecular replacement with crystallographic datasets and a likelihood-based docking tool, EM_placement^76^, with cryo-EM datasets. These determine the correct pose from an initial, unconditional AF2 model. These tools also estimate experimental data parameters for refinement, such as *E*_e_ and *D*_obs_, which represent observed normalized amplitudes and an accounting for measurement error, respectively^77^, and are described further below.

##### Algorithm 1: ROCKET Preprocessing

 **function** PREPROCESSING (ProteinSeq, ReflectionMTZ, HalfMaps)

  $${{\bf{x}}}^{{\rm{initial}}},{{\rm{pLDDT}}}^{{\rm{ref}}},{{\bf{m}}}_{0}^{{\rm{cluster}}\_{\rm{profile}}}=$$ OpenFold_Inference(ProteinSeq)

  pseudo *B*^ref^ = pLDDT_to_pseudo *B*(pLDDT^ref^)

**  if** ReflectionMTZ **then**

   MR_solution, **x**^ref^ = Phasertng_MR(**x**^initial^, ReflectionMTZ)

*   E*_*e*_, *D*_*obs*_ = Phasertng_Preprocess(ReflectionMTZ)

**   return** $${{\bf{x}}}^{\mathrm{ref}},{E}_{e},{D}_{obs},\mathrm{pseudo}\,{B}^{\mathrm{ref}},{{\bf{m}}}_{0}^{\mathrm{cluster}\_\mathrm{profile}},$$$$\,\mathrm{Type}:\mathrm{Crystallographic}$$

**  else if** HalfMaps **then**

**   x**^ref^ = CryoEM_Dock(**x**^initial^, HalfMaps)

*   E*_*e*_, *D*_*obs*_ = CryoEM_ParameterEstimation(HalfMaps)

**   return** $${{\bf{x}}}^{\mathrm{ref}},{E}_{{\rm{e}}},{D}_{\mathrm{obs}},{\mathrm{pseudo}}\,{B}^{\mathrm{ref}},{{\bf{m}}}_{0}^{\mathrm{cluster}\_{\mathrm{profile}}},\,{\mathrm{{Type}}\; :\; {\mathrm{Cryo}}\; -\; {\mathrm{EM}}}$$

**else**

**   return** Error: Missing required data (ReflectionMTZ or HalfMaps)

**end if**

**end function**

#### Refinement algorithm

ROCKET optimizes a linear bias (with scales **w** and offsets **b**) that it applies to the starting MSA cluster profile (${{\bf{m}}}_{0}^{{\rm{cluster}}\_{\rm{profile}}}$) to maximize agreement between an OpenFold prediction (**x**^prediction^) and experimental data (*E*_e_). This agreement is quantified by a data LLG target, ${{\mathcal{L}}}_{\mathrm{LLG}}$, which is combined with an optional positional restraint, ${{\mathcal{L}}}_{L2}$, to yield an overall objective function ${\mathcal{L}}$, described below. The shapes of the **w** and **b** tensors match that of the MSA cluster profile tensor (number of MSA clusters × number of residues × 23)^1^.

##### Algorithm 2: ROCKET Refinement

 **function** REFINEMENT (*E*_e_, *D*_obs_, *N*_iter_, **x**^ref^, pseudo *B*^ref^, ${{\bf{m}}}_{0}^{{\rm{cluster}}\_{\rm{profile}}}$, lr_mul_, lr_add_, *ω*_*L*2_)

  ${\bf{w}}={\rm{ones}}\_{\rm{like}}({{\bf{m}}}_{0}^{{\rm{cluster}}\_{\rm{profile}}})$    ⊳ Initialize multiplicative bias

  ${\bf{b}}={\rm{zeros}}\_{\rm{like}}({{\bf{m}}}_{0}^{{\rm{cluster}}\_{\rm{profile}}})$     ⊳ Initialize additive bias

  optimizer = adam({**w**: lr_mul_, **b**: lr_add_})

**  for** iter = 1…*N*_iter_ **do**

   $${{\bf{m}}}^{{\rm{cluster}}\_{\rm{profile}}}={\bf{w}}\odot {{\bf{m}}}_{0}^{{\rm{cluster}}\_{\rm{profile}}}+{\bf{b}}$$

**   x**^prediction^, pLDDT = OpenFold_Inference(**m**^cluster_profile^)

   pseudo *B* = pLDDT_to_pseudo *B*(pLDDT)

**   x**^array^ = Weighted_Kabsch(**x**^prediction^, **x**^ref^, pseudo *B*^ref^)

**   x**^RBR^ = Rigid_Body_Refinement(**x**^array^, pseudo *B*, *E*_e_, *D*_obs_)

   $${{\mathcal{L}}}_{\mathrm{LLG}}=\,\mathrm{LLG}\,({{\bf{x}}}^{\mathrm{RBR}},\,\mathrm{pseudo}\,B\,,{E}_{{\rm{e}}},{D}_{\mathrm{obs}})$$    ⊳ LLG Targets

   $${{\mathcal{L}}}_{\mathrm{L2}}=\,\mathrm{Weighted}\_\mathrm{L2}\,({{\bf{x}}}^{\mathrm{RBR}},{{\bf{x}}}^{\mathrm{ref}},\mathrm{pseudo}\,{B}^{\mathrm{ref}})$$   ⊳ Weighted L2 loss

   $${\mathcal{L}}={{\mathcal{L}}}_{\mathrm{LLG}}+{\omega }_{\mathrm{L2}}\cdot {{\mathcal{L}}}_{\mathrm{L2}}$$

   $${\mathcal{L}}.\,\mathrm{backward()}$$

   optimizer.step()

**end for**

**  return** **x**^RBR^, **w**, **b**

**end function**

During each ROCKET iteration, we apply a linear bias to the initial cluster profile matrix and then run OpenFold inference to obtain a new prediction, along with its pLDDT confidence values. For crystallography, our current implementation estimates atomic *B* factors at every iteration from pLDDT confidence scores using a previously established heuristic^2^, without explicitly refining them. Specifically, we convert pLDDT values into equivalent r.m.s.d. values, using the empirical relation from Baek et al.^2^, and then to corresponding pseudo *B* factors:

1 $$\,\mathrm{pseudo}\,B\,=\frac{8{\pi }^{2}}{3}{\left[1.5\times {e}^{4\times (0.7-0.01\times \mathrm{pLDDT})}\right]}^{2}$$

At every iteration, we align the newly predicted model to the reference model using a weighted Kabsch alignment (with weights determined using the pseudo *B* factors as described in Eq. 16), followed by rigid-body refinement, elaborated later. We then compute the experimental LLG using the aligned coordinates and the pseudo *B* factors.

In practice, ROCKET runs in two phases: an ‘adventurous’ phase 1 and a ‘fine-tuning’ phase 2. We apply different learning rates for the multiplicative (**w**) and additive (**b**) components of our MSA profile bias and these rates vary across phases. In phase 1, we use higher learning rates (by default, lr_mul_ = 1.0 and lr_add_ = 0.05) and a default low-resolution cutoff of 3 Å. We also incorporate a weighted Cα mean squared distance between the reference model and latest prediction as an *L*_2_ regularization term that quantifies initial model confidence. The weights are computed with the same scheme used for the weighted Kabsch alignment, discussed further below.

2 $${{\mathcal{L}}}_{L2}=w(\,\mathrm{pseudo}\,B)\cdot {\left({{\bf{x}}}^{\mathrm{array}}-{{\bf{x}}}^{\mathrm{ref}}\right)}^{2}$$

with the confidence-based weights *w*(pseudo *B*) defined in Eq. 16 and the final loss for backpropagation:

3 $${\mathcal{L}}={{\mathcal{L}}}_{\mathrm{LLG}}+{\omega }_{L2}{{\mathcal{L}}}_{L2}$$

By default, the *L*_2_ loss weight is *ω*_*L*2_ = 10^−11^. We run phase 1 for three independent traces, each consisting of 100 iterations, and select the model with the best LLG score to proceed to phase 2. The aim of phase 2 is to further fine-tune the structure. By default, we set phase 2 to run for 500 iterations with both lr_mul_ and lr_add_ to 10^−3^ and remove the *L*_2_ loss term (*ω*_*L*2_ = 0). An early stop occurs if the LLG score does not improve by more than 0.1 for 50 consecutive iterations. Furthermore, Supplementary Fig. 19 illustrates the efficacy of phase 1 for avoiding local optima through the example of the c-Abl kinase crystallographic dataset (PDB 3PYY).

The pseudo *B* approximation can limit accuracy by not capturing finer structural details. Moreover, geometric validation indicates that outputs from the iterative optimization have more bond outliers and steric clashes than stricter refinement protocols typically allow (Supplementary Fig. 5f). To address these limitations, we append a short standard local refinement step using phenix.refine^16^ after iterative OpenFold inference. Analogous to AMBER relaxation in standard AF2 pipelines^1^, this step further optimizes geometry (Supplementary Fig. 5) and displacement parameters, polishing the final model’s overall quality.

#### Implementation

ROCKET is implemented in PyTorch 1.12.1 as an extension of the OpenFold system. It currently uses the monomer version of OpenFold with AF2 model_1 weights to maintain consistency with AF2’s data splits. This allows for prediction and refinement of crystallographic datasets containing a single chain in the asymmetric unit or one domain (at a time) in a cryo-EM complex.

### Crystallographic LLG targets

For crystallographic datasets, we use the LLG on intensity (LLGI) target introduced in a previous study^77^, which can be expressed as follows, for acentric and centric reflections:

4 $$\begin{array}{l}{{\rm{LLGI}}}_{{\rm{a}}}({E}_{{\rm{e}}};{E}_{{\rm{C}}})={\rm{ln}}\,[\frac{{p}_{{\rm{a}}}({E}_{{\rm{e}}};{E}_{{\rm{C}}})}{{p}_{{\rm{a}}}({E}_{{\rm{e}}})}]\\ \,\,\,\,\,\,\,\,\,\,\,\,\,\,\,\,\,\,\,\,\,\,\,\,\,\,\,\,\,\,\,\,\,\,\,\,\,={\rm{ln}}\,[{p}_{{\rm{a}}}({E}_{{\rm{e}}};{E}_{{\rm{C}}})]-{\rm{ln}}\,[{p}_{{\rm{a}}}({E}_{{\rm{e}}})]\end{array}$$

5 $$\begin{array}{l}{{\rm{LLGI}}}_{{\rm{c}}}({E}_{{\rm{e}}};{E}_{{\rm{C}}})={\rm{ln}}\,[\frac{{p}_{{\rm{c}}}({E}_{{\rm{e}}};{E}_{{\rm{C}}})}{{p}_{{\rm{c}}}({E}_{{\rm{e}}})}]\\ \,\,\,\,\,\,\,\,\,\,\,\,\,\,\,\,\,\,\,\,\,\,\,\,\,\,\,\,\,\,\,\,\,\,\,\,\,={\rm{ln}}\,[{p}_{{\rm{c}}}({E}_{{\rm{e}}};{E}_{{\rm{C}}})]-{\rm{ln}}\,[{p}_{{\rm{c}}}({E}_{{\rm{e}}})]\end{array}$$

with $${p}_{{\rm{a}}}\left({E}_{{\rm{e}}}\right)=2{E}_{{\rm{e}}}\exp \left(-{E}_{{\rm{e}}}^{2}\right)$$, $${p}_{{\rm{c}}}\left({E}_{{\rm{e}}}\right)={\left(\frac{2}{\pi }\right)}^{1/2}\exp \left(-\frac{{E}_{{\rm{e}}}^{2}}{2}\right)$$ and

6 $$\begin{array}{l}{p}_{{\rm{a}}}({E}_{{\rm{e}}};{E}_{{\rm{C}}})=\frac{2{E}_{{\rm{e}}}}{1-{D}_{\mathrm{obs}}^{2}{\sigma }_{{\rm{A}}}^{2}}\exp \left[-\frac{{E}_{{\rm{e}}}^{2}+{({D}_{\mathrm{obs}}{\sigma }_{{\rm{A}}}{E}_{{\rm{C}}})}^{2}}{1-{D}_{\mathrm{obs}}^{2}{\sigma }_{{\rm{A}}}^{2}}\right]\\ \,\,\,\,\,\,\,\,\,\,\,\,\,\,\,\,\,\,\,\,\,\,\,\,\,\,\,\times {I}_{0}\left(\frac{2{D}_{\mathrm{obs}}{\sigma }_{{\rm{A}}}{E}_{{\rm{e}}}{E}_{{\rm{C}}}}{1-{D}_{\mathrm{obs}}^{2}{\sigma }_{{\rm{A}}}^{2}}\right)\end{array}$$

7 $$\begin{array}{l}{p}_{{\rm{c}}}({E}_{{\rm{e}}};{E}_{{\rm{C}}})={\left[\frac{2}{\pi (1-{D}_{\mathrm{obs}}^{2}{\sigma }_{{\rm{A}}}^{2})}\right]}^{1/2}\exp \left[-\frac{{E}_{{\rm{e}}}^{2}+{({D}_{\mathrm{obs}}{\sigma }_{{\rm{A}}}{E}_{{\rm{C}}})}^{2}}{2(1-{D}_{\mathrm{obs}}^{2}{\sigma }_{{\rm{A}}}^{2})}\right]\\ \,\,\,\,\,\,\,\,\,\,\,\,\,\,\,\,\,\,\,\,\,\,\,\,\,\,\,\times \cosh \left(\frac{{D}_{\mathrm{obs}}{\sigma }_{{\rm{A}}}{E}_{{\rm{e}}}{E}_{{\rm{C}}}}{1-{D}_{\mathrm{obs}}^{2}{\sigma }_{{\rm{A}}}^{2}}\right)\end{array}$$

where *p*(*x*; *y*) denotes the conditional probability of *x* given *y*.

As defined previously^77^, *E*_e_ is the ‘effective’ observed normalized amplitude, *E*_C_ is the normalized structure factor amplitude calculated from the predicted model in a differentiable manner using SFCalculator with solvent correction^34^, *D*_obs_ encodes the reduction in correlation between true and effective normalized structure factors arising from experimental error and *σ*_A_ is a resolution-dependent factor that encodes the reduction in correlation between the true and calculated normalized structure factors arising from model error.

We refine *σ*_A_ in resolution bins^78^ at every ROCKET iteration using the Newton–Raphson optimization method^79^. The LLG of the observed effective amplitudes, *E*_e_, given the calculated amplitudes, *E*_C_, is maximized by refining *σ*_A_. The derivative of the LLG with respect to *σ*_A_ for each resolution bin is given by

8 $$\frac{\partial \mathrm{LLG}}{\partial {\sigma }_{{\rm{A}}}}=\mathop{\sum }\limits_{i}\frac{\partial \,{\mathrm{ln}}\,p\left({E}_{{\rm{e}},i};{E}_{{\rm{C}},i},{\sigma }_{{\rm{A}}}\right)}{\partial {\sigma }_{{\rm{A}}}}$$

where *i* is an index over observations. The second derivative is given by

9 $$\frac{{\partial }^{2}\,\mathrm{LLG}}{\partial {\sigma }_{{\rm{A}}}^{2}}=\mathop{\sum }\limits_{i}\frac{{\partial }^{2}\,{\mathrm{ln}}\,p\left({E}_{{\rm{e}},i};{E}_{{\rm{C}},i},{\sigma }_{{\rm{A}}}\right)}{\partial {\sigma }_{{\rm{A}}}^{2}}$$

We obtain the updated value of *σ*_*A*_ using a Newton step:

10 $${\sigma }_{{\rm{A}}}^{\mathrm{new}}={\sigma }_{{\rm{A}}}- \left( {\frac{{\partial }^{2}{{\mathcal{L}}}_{\mathrm{LLG}}}{\partial {\sigma }_{{\rm{A}}}^{2}}}\right)^{-1}\frac{\partial {{\mathcal{L}}}_{\mathrm{LLG}}}{\partial {\sigma }_{{\rm{A}}}}$$

The value of *σ*_A_ is constrained within [0.015, 0.99] to maintain physical relevance and stability during refinement. For all LLG and *σ*_A_ estimates we use the working set of reflections (we find that using the working set for *σ*_A_ refinement does not lead to any meaningful overfitting with ROCKET; Supplementary Fig. 18). We keep a test set of reflections for final *R*_free_ calculation after conventional refinement.

### LLG target and noise modeling for cryo-EM

For cryo-EM data, we follow the method outlined previously^76^ to dock the initial prediction into the experimental map. ROCKET works with a sphere surrounding the model, with contributions from any other fixed components within that sphere being accounted for. We model the signal and noise in Fourier space to account for directional and resolution-dependent variations in data quality. The signal is derived from correlations between Fourier terms of the experimental half-maps, while the noise is deduced from their differences^53^.

For a single Fourier term, the LLG is given by

11 $$\begin{array}{l}{\mathrm{LLG}}_{\mathrm{cryo}}=\frac{2\cdot {D}_{\mathrm{obs}}\cdot {\sigma }_{A}\cdot {E}_{{\rm{e}}}\cdot {E}_{\mathrm{calc}}\cdot \cos (\Delta \phi )}{1-{D}_{\mathrm{obs}}^{2}{\sigma }_{A}^{2}}\\ \,\,\,\,\,\,\,\,\,\,\,\,\,\,\,\,\,\,\,\,-\frac{{D}_{\mathrm{obs}}^{2}{\sigma }_{A}^{2}({E}_{{\rm{e}}}^{2}+{E}_{\mathrm{calc}}^{2})}{1-{D}_{\mathrm{obs}}^{2}{\sigma }_{A}^{2}}-\mathrm{ln}\,\left(1-{D}_{\mathrm{obs}}^{2}{\sigma }_{A}^{2}\right)\end{array}$$

where *E*_e_ and *E*_calc_ are the normalized amplitudes of the observed and calculated Fourier terms, = *ϕ*_calc_ − *ϕ*_obs_ is the phase difference between these Fourier terms and *D*_obs_ and *σ*_*A*_ are analogous to their crystallographic counterparts.

We compute *σ*_A_ for each resolution bin as described previously^53^, using observed normalized amplitudes (*E*_e_), calculated normalized amplitudes (*E*_calc_) and the phase difference Δ*ϕ* = *ϕ*_calc_ − *ϕ*_obs_.

For the cryo-EM cases, which are not affected by phase bias, we update residue-level *B* factors at each iteration using a conversion that is informed by local RSCC to the experimental map.

### Cryo-EM map resolution degradation

For testing, it is invaluable to have low-resolution cryo-EM maps for which the ground truth is known from a corresponding higher-resolution map. Simple Fourier filtering can remove high-resolution information but the resulting data are much higher in quality at the new resolution limit than one would encounter with real data.

One approach, used for instance in testing the *Q*-score algorithm^80^, is to make reconstructions with progressively fewer particle images. This approach was used to generate lower-resolution versions of the GroEL:GroES–ATP complex, using cryoSPARC^81^ to reprocess data obtained from EMPIAR-11481. Subsets of particles used in the original reconstruction were selected randomly: a reconstruction with *C*_7_ symmetry using 45,174 particles yielded a map at 2.9-Å resolution, while maps at the lower resolutions of 4.9 Å and 6.8 Å were produced using 310 and 247 particles, respectively.

A second approach, in which independent complex random noise is added to the half-map Fourier terms, is much faster and allows finer control of resolution limits. Implementing this approach required first defining targets for the FSC curves that would be expected at different resolution limits. This was achieved by devising a functional form for an equation that could fit a wide variety of FSC curves in the EM Data Bank. The underlying idea for the functional form is that the variation of FSC with resolution is controlled by the relative size of the signal and noise powers at zero scattering angle and the difference in how those powers fall off with resolution.

12 $${\rm{FSC}}=\frac{r}{r+\exp (\Delta B| s{| }^{2}/4)}$$

where *r* is the ratio between the signal and noise powers at *s* = 0 and Δ*B* is the difference in the falloff *B* factors for signal and noise.

FSC curves were downloaded for all the EM Data Bank entries where half-map data and author-supplied FSC curves were deposited in 2024. These were ordered by nominal resolution and every tenth entry was taken, yielding 458 cases. Good fits were obtained for most FSC curves by optimizing the values of *r* and Δ*B;* the best-fit resolution (*s*_max_ = 1/*d*_min_) was defined as the point where the fitted FSC curve was equal to 0.143. Then functional forms for *r* and Δ*B* in terms of *s*_max_ were fitted.

13 $$r=34.4680\,\exp (3.00533{s}_{\max }+4.27895{s}_{\max }^{2})$$

14 $$\Delta B=17.1158+12.0213/{s}_{\max }+21.3225/{s}_{\max }^{2}$$

To match the target FSC curve in a particular case, the Fourier terms are divided into resolution shells. In each shell, the signal and noise powers are determined and then the amount of noise required to match the desired FSC is computed and added to the half-map Fourier terms. Finally, the half-maps are computed using only the Fourier terms to *s*_max_.

### Weighted Kabsch alignment and rigid-body refinement

As stated above, we use the initial prediction from OpenFold to run Phasertng (or EM_placement for cryo-EM) for molecular replacement. For every iteration, we align the OpenFold model to the reference molecular replacement solution before computing the relevant LLG score. This alignment is performed by first solving the following minimization problem with the Kabsch algorithm^82^:

15 $$L(C)=\frac{1}{2}\mathop{\sum }\limits_{i=1}^{n}{w}_{i}{\left\Vert {{\bf{x}}}_{i}^{{\rm{ref}}}-C{{\bf{x}}}_{i}^{{\rm{prediction}}}\right\Vert }^{2}$$

where *C* denotes the rotation–translation matrix, **x**^ref^ and ${{\bf{x}}}_{i}^{{\rm{prediction}}}$ are corresponding atomic coordinates of atom *i* in the reference and moving model, respectively, and *w*_*i*_ denotes positional weights. Only Cα atoms are included in the alignment and their weights are determined empirically from the pseudo *B* values of the reference structure (the first, unconditioned OpenFold prediction). Specifically, for each residue,

16 $$w(\,\mathrm{pseudo}\,B)=\left\{\begin{array}{ll}1.0 & \mathrm{pseudo}\,B\,\le 11.5\\ 1.0-0.5\times \frac{\,\mathrm{pseudo}\,B\,-11.5}{40.0-11.5} & 40.0\ge \,\mathrm{pseudo}\,B\, > 11.5\\ 0.5\exp (-\sqrt{\,\mathrm{pseudo}\,B\,-40.0}) & \mathrm{pseudo}\,B\, > 40.0\end{array}\right.$$

Translation vectors are determined by the vector difference of weighted center of mass of Cα atoms in the reference and moving models; then, the rotation matrices are estimated with Kabsch algorithm. Once the alignment is completed, a subsequent rigid-body refinement is performed through gradient optimization of the LLG target:

17 $${C}^{* }=\arg \mathop{\max }\limits_{C}\mathrm{LLG}({E}_{{\rm{o}}},{\bf{x}},C)$$

where **x** represents the model coordinates after Kabsch alignment. When the predicted aligned error matrix from AF2 can be used automatically to split proteins into domains, ROCKET performs domain-specific alignments to make the best use of gradient information from the LLG.

### Multichain dataset handling

ROCKET can readily handle monomeric protein predictions in its current form. We also demonstrated refinement of crystallographic datasets that contain two chains in the asymmetric unit for the kinase datasets (PDB 3PYY in Supplementary Fig. 6 and PDB 7DT2 in Supplementary Fig. 16). For these cases, we included the second chain present in the asymmetric unit as a fixed Fourier contribution in the likelihood calculation but excluded it from refinement.

General multichain refinement requires integrating ROCKET with a multimeric model such as OpenFold-Multimer. We present a first demonstration on ZPD (Fig. 4), where chains A–C are predicted and refined simultaneously using OpenFold-Multimer weights at inference time. ZPD is a relatively small multimeric complex, allowing this demonstration within ROCKET’s current memory constraints.

### ZPD sample preparation and structure determination

Chicken ZPD was prepared following an established procedure^70^, except that a HEPES buffer (H0887, Sigma) supplemented with protease inhibitors (protease inhibitor cocktail set III, EDTA-free; Calbiochem) was used. On the basis of densitometry using BSA bands as standards (Supplementary Fig. 20a), the yield of ZPD was estimated at ~0.35 mg from ~70 mg wet weight of chicken egg coat. ZPD was notably concentrated in the crude ZPD preparation (reaching ~0.70 mg ml^−1^), albeit with residual contamination of ZP1 and ZP3 (Supplementary Fig. 20).

Data processing was carried out using cryoSPARC v.4.7.0. Following patch motion and patch contrast transfer function estimation, filaments were initially traced in a subset of 2,000 micrographs. Particles were extracted with a box size of 512 pixels and Fourier-cropped to 128 pixels for two-dimensional (2D) classification, generating initial templates for subsequent template-based filament tracing. After several rounds of tracing using well-defined classes, a total of 1,113,548 particles were extracted and subjected to iterative 2D classification to remove low-quality particles. Ab initio reconstruction was then performed, followed by heterogeneous and homogeneous refinement. To further resolve particle heterogeneity, 3D classification with five classes was conducted using a principal component analysis-based approach. From this, a final dataset of 317,745 unbinned particles was selected. Helical parameters were determined by indexing the power spectrum^83^ generated from cryoSPARC’s ‘average power spectra’ job and compared to power spectra of potential models using SPIDER^84^. Initial helical twist 126.8° and rise of 69.7-Å values obtained from this analysis were used as input for helical refinement in cryoSPARC, which refined them to a twist of 120.8° and a rise of 71.4 Å. A maximum symmetry order of 3 was applied during reconstruction, resulting in a map with a nominal resolution of 8.6 Å. A single-particle processing strategy was applied to the second, higher-resolution dataset. A total of 2,031,564 particles were extracted with a box size of 512 pixels and Fourier-cropped to 128 pixels. Iterative 2D classification, combined with heterogeneous refinement including multiple noise classes, was performed to clean the dataset. Selection of good classes after 3D classification resulted in a final subset of 498,339 unbinned particles, which were further subjected to homogeneous and nonuniform refinement. A focused mask was then applied to the central region of the map for local refinement, producing a reconstruction with a nominal resolution of 4.6-Å resolution. Data collection statistics for the two ZPD datasets are reported in Supplementary Tables 2 and 3.

For non-ROCKET modeling of ZPD, a local installation of AF3 (ref. ^9^) was used to predict a minimal filament fragment encompassing a full protein chain and two half ones (as previously done for uromodulin^72^, as well as the ZP1–ZP3 complex^69^). The top-scoring model (ranking score 0.8) was then fitted into a version of the ZPD low-resolution map postprocessed by EMReady2 (ref. ^71^) through rigid-body docking in Chimera^85^, followed by flexible fitting with Namdinator^86^, trimming of nonresolved terminal residues and fitting of clear *N*-glycan densities in Coot^87^. The resulting model was finally subjected to positional real-space refinement using noncrystallographic symmetry (NCS) constraints and increased nonbonded interaction weights, followed by atomic displacement parameter refinement against the unsharpened map with phenix.real_space_refine^88^. The coordinates refined against the low-resolution data were used as a starting point for extending the model with an additional EGF and ZP-N domain fragment from a fourth ZPD subunit; after rigid-body docking into the medium-resolution map and manual editing of N termini and glycans, the resulting seven-domain model was flexibly fitted using the cryo_em_minimizer script of the cg2all package^89^. Refinement was initially performed with phenix.real_space_refine as described above; subsequently, the model was refined against the medium-resolution half-maps using the REFMAC Servalcat task of CCP-EM Doppio^90,91^ and applying global NCS restraints, ProSMART^92^-generated self-restraints and an increased weight of nonbonded interactions (‘vdwr 2’). Model geometry and map-fitting parameters were computed using the comprehensive validation tool of PHENIX^93^. Helical indexing of the medium-resolution single-particle map of ZPD, performed with HI3D^94^, yielded helical parameters of twist = 115° and rise = 71 Å.

### Data visualization

Visualization of PDB structures and experimental densities was performed with PyMOL (Schrödinger)^95^ and Moorhen^96^. Structure validation was performed with MolProbity^97^.

### Reporting summary

Further information on research design is available in the Nature Portfolio Reporting Summary linked to this article.

## Online content

Any methods, additional references, Nature Portfolio reporting summaries, source data, extended data, supplementary information, acknowledgements, peer review information; details of author contributions and competing interests; and statements of data and code availability are available at 10.1038/s41592-026-03047-4.

## Supplementary information

Supplementary Information Supplementary Text, Figures 1–20, Tables 1–3 and References.

Reporting Summary

## Data Availability

Datasets are made available for tutorials and further experiment on Zenodo (10.5281/zenodo.15084557)^98^. Cryo-EM maps and atomic coordinates of the ZPD filament have been deposited in EMDB and PDB with accession codes 28YJ and EMD-56971, respectively.

## Code availability

All ROCKET code and target functions are available from GitHub (https://github.com/alisiafadini/ROCKET) and the v.0.1.0 used for the paper is archived on Zenodo (10.5281/zenodo.15084557)^98^. Documentation and tutorials can be found online (https://rocket-9.gitbook.io/rocket-docs).
