# Supplementary material for: AlphaFold as a prior: experimental structure determination conditioned on a pretrained neural network
Source: Nat Methods. 2026 Apr 1;23(4):785–95. doi: 10.1038/s41592-026-03047-4 (PMC13124595; doi:10.1038/s41592-026-03047-4)
Supplement: Supplementary file 1 — Supplementary Text, Figures 1–20, Tables 1–3 and References. [file 41592_2026_3047_MOESM1_ESM.pdf]

nature methods

Supplementary information https://doi.org/10.1038/s41592-026-03047-4

AlphaFold as a prior: experimental structure determination conditioned on a pretrained neural network

In the format provided by the authors and unedited

Supplementary Information Contents

Supplementary Text 2

CryoEM PPM1H/phospho-Rab8a Sample Preparation, Data Collection and Processing . . 2

Construct design and PPM1H Dephosphorylation Assay 2

Supplementary Figures 3

Supplementary Tables 23

Supplementary References 25

Supplementary Text

CryoEM PPM1H/phospho-Rab8a Sample Preparation, Data Collection and Processing

Purified phosphorylated Rab8a (pRab8a, 20kDa, phospo-Thr72) and PPM1H phosphatase (50kDa) were expressed and purified separately using previously established protocols in Tris-Cl buffer (20mM, pH 7.6), NaCl (150mM), $MgCl_2$ (5mM) and 1mM DTT[9]. To generate a substrate-trapped complex, $CaCl_2$ (50mM final concentration) was added to the purified proteins. Approximately 2.5mg of PPM1H and 1.0mg of pRab8a were mixed together, and the volume was adjusted to 1mL. Ca^{2+} and Zn^{2+} ions are known to inhibit the catalytic activity of the metal-dependent PPM phosphatases, which require either Mg^2+ or Mn^{2+} at the active site[3]. The complex was immediately loaded onto a Superdex 200 (10/300) column buffered with 20mM Tris-Cl (pH 7.6), 150mM NaCl, 1mM DTT and 50mM $CaCl_2$.

The gel filtration peak corresponding to the PPM1H/pRab8a complex was collected and immediately used for cryo-EM grids preparation. 4μL of complex at 0.3mg/mL was loaded onto a freshly glow-discharged Quantifoil holey carbon film on gold support, R 1.2/1.3, 300 mesh, and plunged in liquid ethane using a VitroBot Mark IV, at 4°C and 100% humidity. Movies were collected on Titan Krios operating at 300 keV equipped with a Flacon4i direct electron detector. Collection was performed in counting mode at a 0.74 Å, with a total dose of 50e/ Å^2 over 40 frames, and defocus range between -0.5 and -2μm. Data were processed using CryoSPARC version 4.5.3[7]. Patch CTF estimation was performed following motion correction. Poor-quality micrographs were removed from the downstream analysis. Particles were picked by Topaz Picker. Extracted particles were subjected to multiple rounds of 2D classification, showing one main orientation. Good 2D classes were used to generate three initial models. Extensive 3D classification generated elongated maps due to significant orientation bias. ModelAngelo v1.0.13 was used with default settings to generate the model in Fig. S12.

Construct design and PPM1H Dephosphorylation Assay

The cDNA corresponding to PPM1H-βMdel was designed by removing residues 481-494 from WT PPM1H that has been previously described[9]. These were replaced by two serine residues to enable deletion of the β-motif without compromising the fold of the enzyme. For design of PPM1H-Jflap, residues 304-413 of PPM1H were replaced by residues 295-404 of PPM1J. In addition, for all constructs, an N-terminally truncated form of the enzyme (1-32) along with deletion of a large flexible loop (188-226) and replacement by a flexible linked was designed. Therefore, constructs WT PPM1H, PPM1H-Jflap and PPM1H-bMdel comprise residues 33-514 with the loop deletion to facilitate high purity and stability as described[9]. The cDNAs for PPM1H-Jflap and PPM1H-bMdel cDNA were synthesized by Gesncript with optimized E.coli codons and inserted into pET-28a-TEV vector at the NdeI/BamH1 site. Expression and purification protocols have previously been described, with the final step being gel filtration chromatography[9]. The purity of the proteins was $> 90\%$ as evidenced by analytical SDS-PAGE gels visualized by Coomassie Blue.

Dephosphorylation assays were performed in triplicate with 15µM phospho-Rab8a in a total reaction volume of 30µl buffer (20mM Tris-HCl pH 8, 150mM NaCl, 1mM DDT, 5 mM $MgCl_2$). The reaction was initiated by addition of 30nM PPM1H and incubated for 30min at room temperature. The reaction was terminated by addition of 10µl 4X SDS sample buffer. The samples were subjected to Phos-Tab gel electrophoresis (FUJIFILM) and gels stained with Coomassie blue staining.

Supplementary Figures

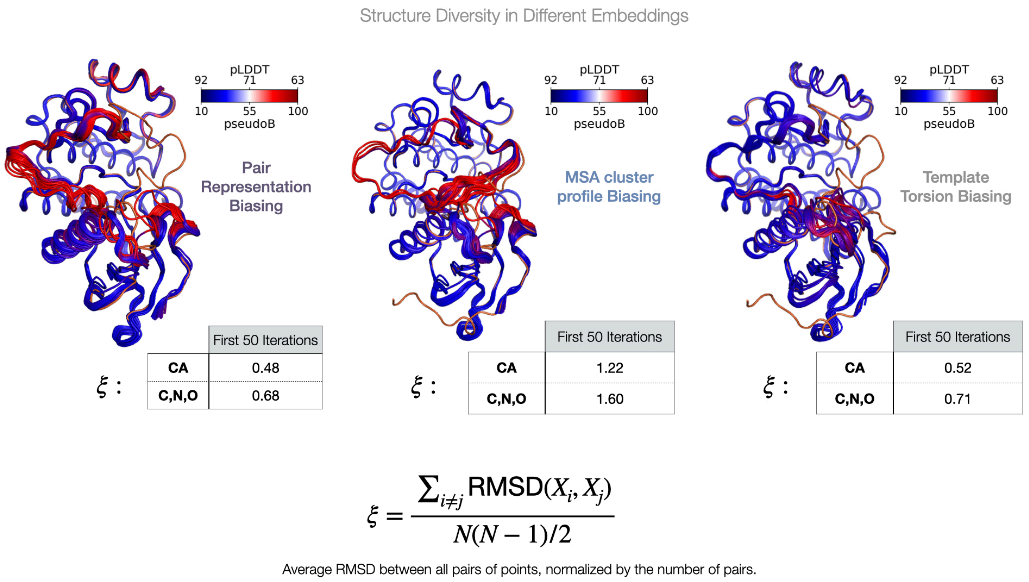

Fig. S1 ROCKET Optimization in Different AlphaFold2 Embeddings. We performed data-guided inference-time optimization, as described in the main text, in alternative embeddings. We found that optimizing MSA cluster profiles provides the highest structural diversity along search trajectories. We calculated an average RMSD (ξ) between all pairs of structures, normalized by the number of pairs for the first 50 structures in a ROCKET phase 1 refinement run. Diversity is much higher when biasing the MSA cluster profile ($\xi = 1.22$ versus $\xi = 0.48$ and $\xi = 0.52$ when biasing the pair and template torsion representations, respectively).

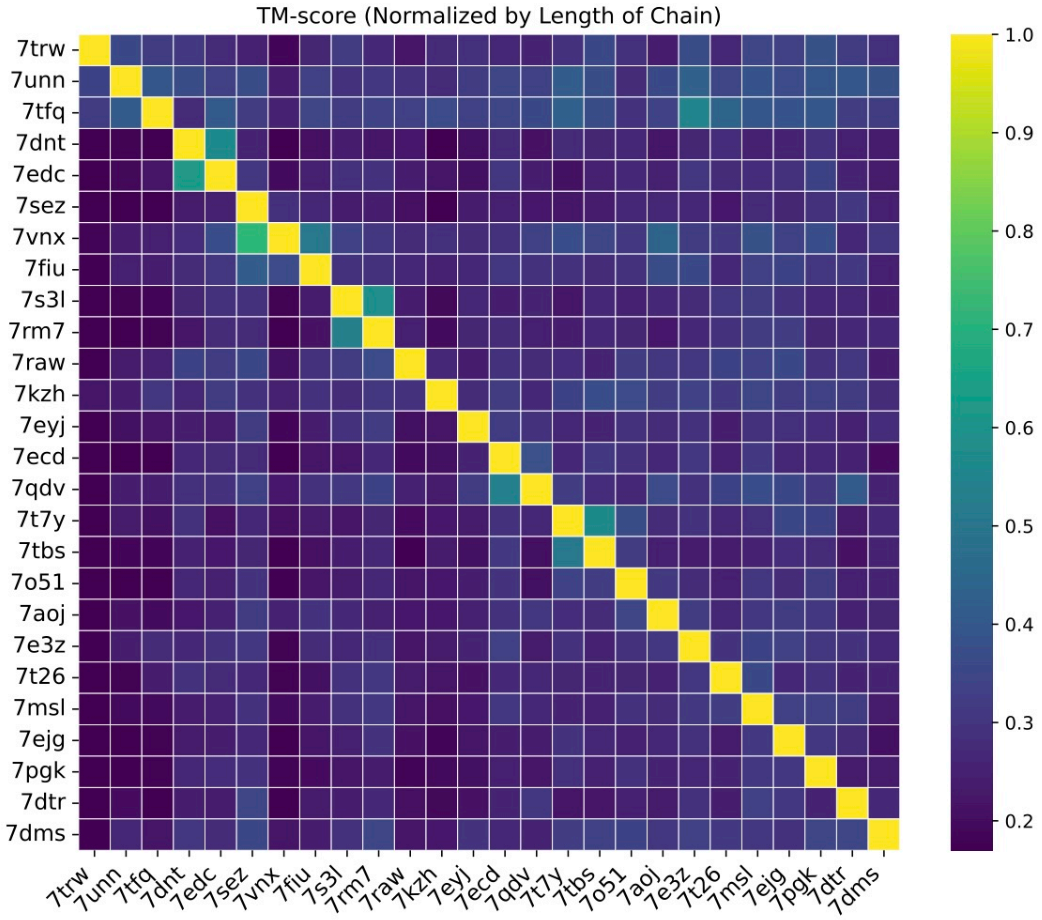

Fig. S2 Fold Diversity in High-Resolution Benchmark Dataset. Template Modeling (TM) scores for the 27 structures present in our crystallographic benchmark dataset, identified through their PDB ID. The TM-score evaluates how well two structures align in three-dimensional space, independent of sequence identity, with TM-score closer to 1 indicating high similarity in the two folds. The low TM-scores between structures reflect their fold diversity. These structures were all released after the training of the AF2 weights used here and were solved by the single-wavelength anomalous diffraction (SAD) method, suggesting that it was challenging to find structural homologs in the PDB for molecular replacement. We therefore expect limited leakage from the original AF2 training set.

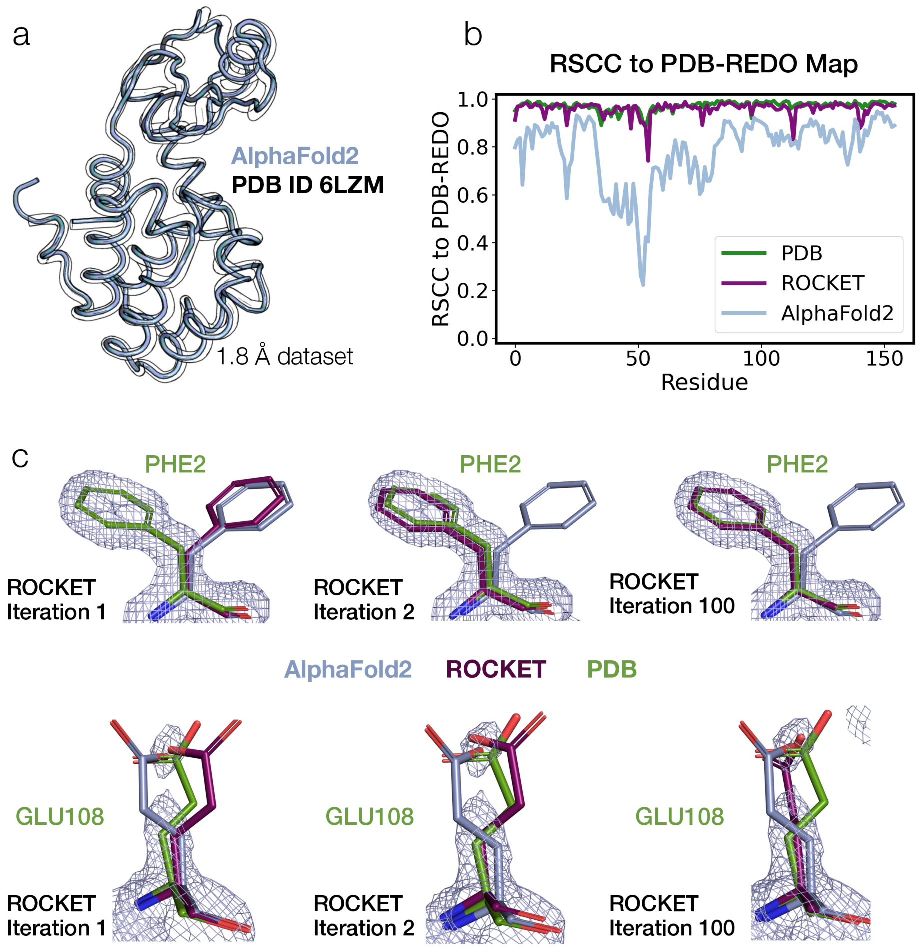

Fig. S3 MSA Cluster Profile Fine-Tuning Enables Both Backbone and Sidechain Refinement. Example of ROCKET refinement for a bacteriophage T4 lysozyme dataset (related PDB ID 6LZM), which differs from the AF2 prediction, particularly in the orientation of its upper domain (a). The PDB REDO structure is shown in transparent outlines. (b) ROCKET can refine the prediction to the same quality as the deposited structure. (c) When the data support a clear conformation (*e.g.*, for PHE2), ROCKET is able to find different rotameric configurations and gradually fine-tune the sidechain position. For GLU108, where the data are noisy, ROCKET explores different conformations and, in this case, settles for placing a carboxylate oxygen atom in the available density.

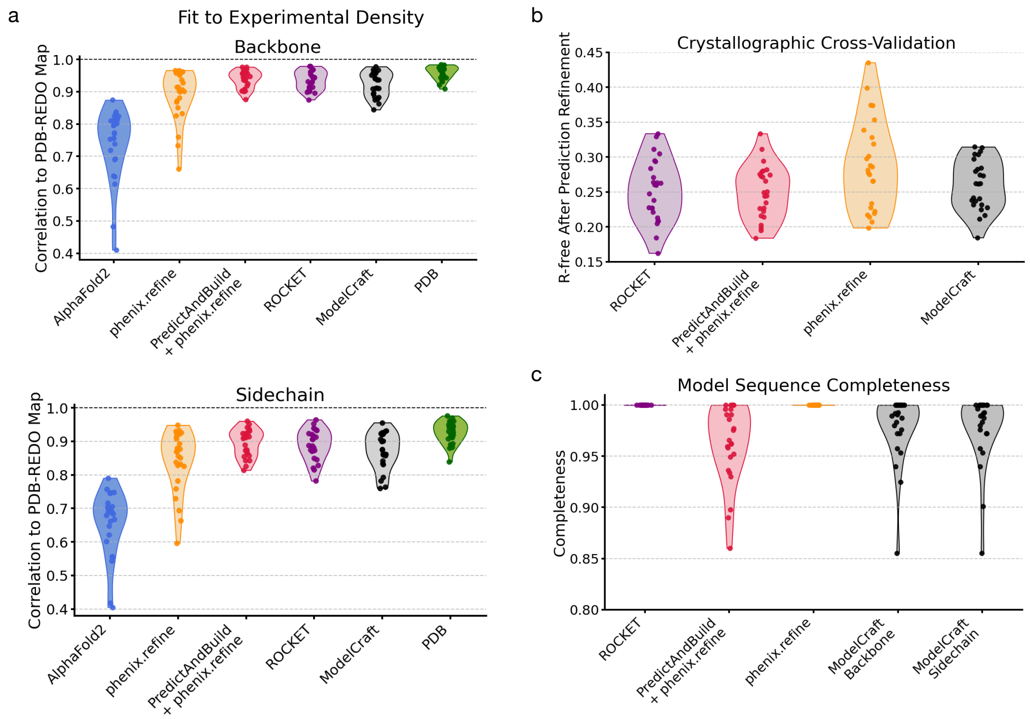

Fig. S4 High-Resolution X-ray Crystallography Benchmark. AF2 inference, conventional refinement (phenix.refine), iterative AF2 inference with external rebuilding (PredictAndBuild + phenix.refine), internal iterative AF2 inference and rebuilding (ROCKET), and de novo model building (ModelCraft) are compared. (a) Real-space Pearson correlation coefficient (RSCC) values of electron density maps derived from model structures vs. experimental amplitudes and unbiased phases from PDB-REDO. PDB-REDO maps are expected to have a favorable phase bias for deposited PDB models, artificially increasing their reported RSCCs. (b) Crystallographic R-free values for models placed by molecular replacement and refined using different methods. (c) Final model sequence completeness for the different methods used.

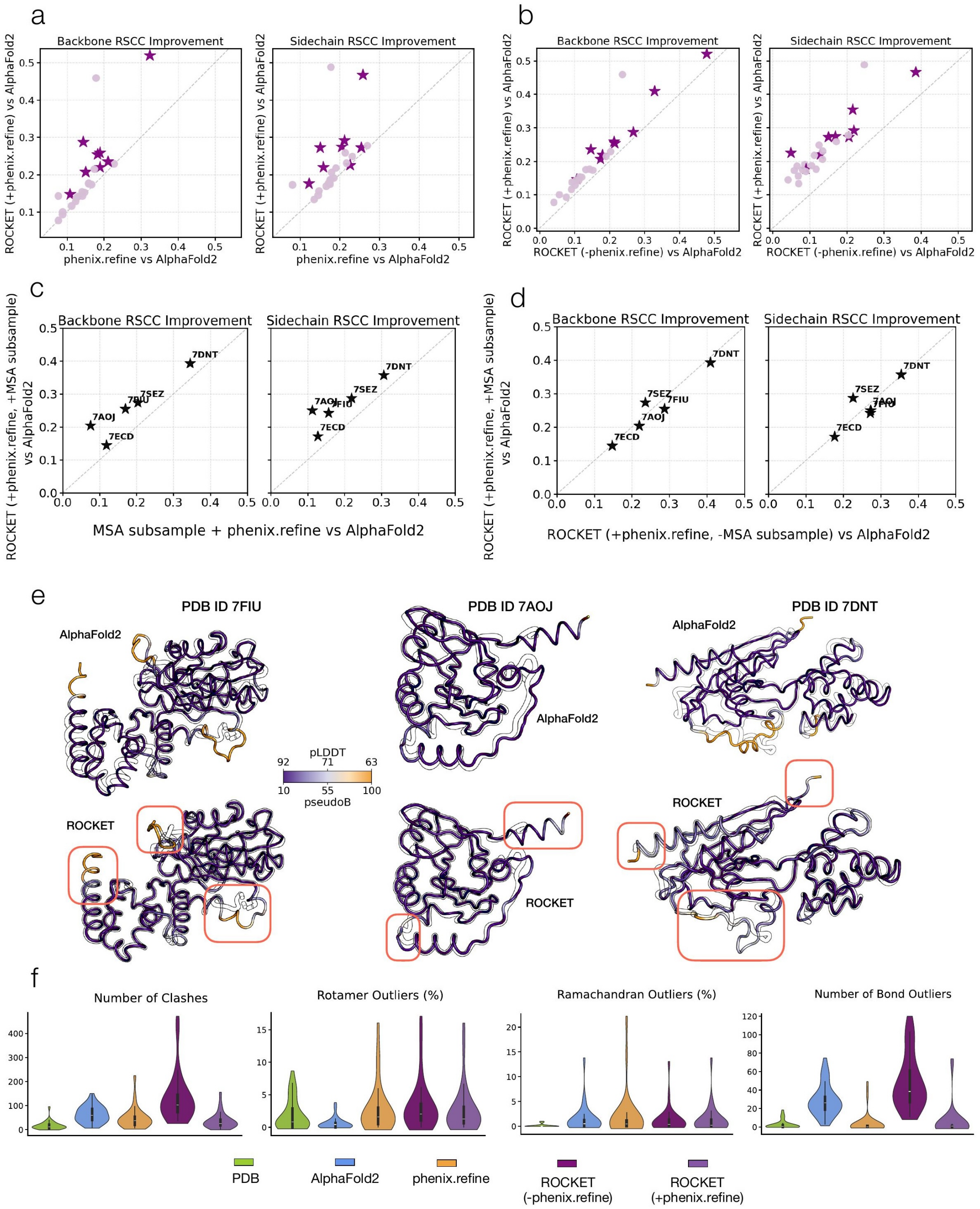

Fig. S5 Internal Ablation of ROCKET Refinement Protocols. (a) Improvements in real-space Pearson correlation coefficient (RSCC) values for backbone and sidechains for phenix.refine and full ROCKET refinement, when compared to initial AF2 models. Same datasets and symbols as in Fig. 2a. RSCC calculations include residues for which AF2 and ROCKET report low confidence. (b) Breakdown of the incremental RSCC improvement from the initial AF2 prediction to the ROCKET structures before and after the phenix.refine step. (c-d) RSCC improvements based on combinations of phenix.refine, MSA subsampling+scoring, and internal ROCKET refinement for the 5 most difficult cases in the high-resolution benchmark (final RMSD between ROCKET model and deposited model > 1.0). (e) Three such examples, PDB IDs 7FIU, 7AOJ and 7DNT, highlight models with stretches of sequence that are not supported by strong density and are not improved by ROCKET. For the 7AOJ and 7DNT, ROCKET also fails to flip small loops of the kind discussed in Fig. S16. Importantly, the regions where the ROCKET models are not supported by density are characterized by low AF2 confidence (low pLDDT) for those residues. (f) Geometric validation after refinement by different conditions, carried out using MolProbity [11] for the 27 high-resolution crystallographic test cases. Box plot elements: Box spans 25th to 75th percentile (interquartile range, IQR); center line represents the median; whiskers extend to 1.5×IQR or to the minimum/maximum value if within that range; points beyond whiskers are outliers.

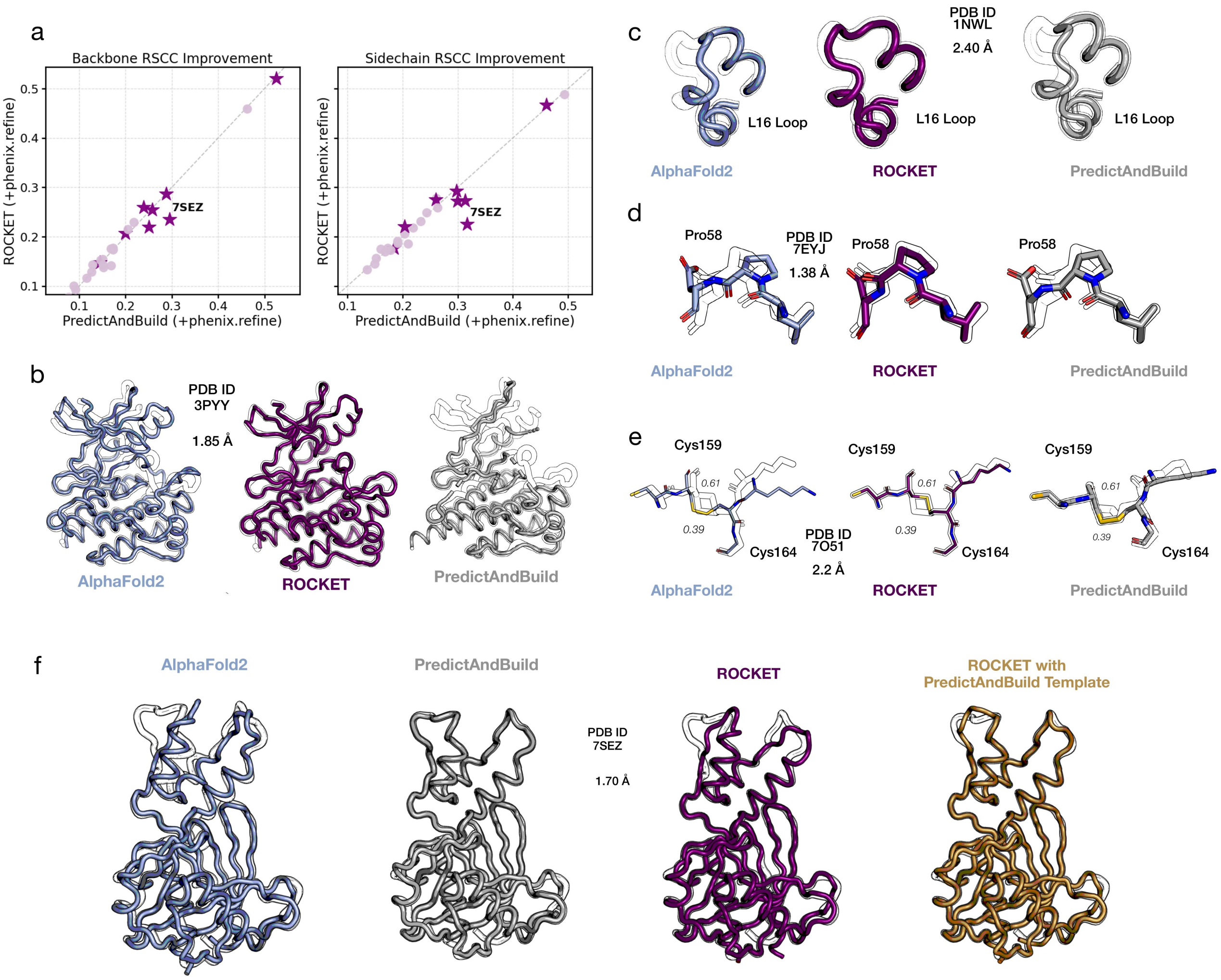

Fig. S6 Comparison between ROCKET and PredictAndBuild. (a) Improvements in real-space Pearson correlation coefficient (RSCC) values for backbone and sidechains for PredictAndBuild and full ROCKET refinement, when compared to initial AF2 models. RSCC calculations include residues for which AF2 and ROCKET report low confidence. (b-c) PredictAndBuild often chops low-confidence regions and flexible loops, leaving them for manual rebuilding. ROCKET can handle these automatically. This is demonstrated for the ligand-induced rearrangements of the activation and P-loops in human c-Abl kinase (PDB ID 3PYY — b) and for the ligand induced peptide flip in the L16 loop of protein tyrosine phosphatase PTP-1B (PDB ID 1NWL — c). Ligand molecules (not shown) are not considered by ROCKET, as its refinement depends purely on experimental density without explicit modeling of protein-ligand interactions. (d-e) Two examples of bond rearrangements applied by ROCKET that PredictAndBuild does not access. The first is a peptide flip in the refinement of *E. coli* nucleoside phoshorylase (PDB ID 7YEJ, (d)). The second is a switch in a disulphide bond in the structure of thaumatin from *T. daniellii* (PDB ID 7O51, (e)). Two alternate conformations are present in the deposited structure, with refined occupancies of 0.39 and 0.61. ROCKET builds the conformation with highest occupancy. PDB REDO models are shown in black outlines. (f) We discuss in the main text and further in Fig. S16 that ROCKET can struggle to flip small loops containing long sidechain residues. The refinement of the Vaccinia Virus decapping enzyme D9 (PDB ID 7SEZ) is an example of this, where ROCKET is unsuccessful in modeling two small loops in the upper domain, while PredictAndBuild converges to the correct backbone. We show the complementarity of the two approaches by running ROCKET with the PredictAndBuild structure provided as a template during OpenFold inference and improving the final output. We also note that ROCKET MSA subsampling and subsequent likelihood scoring can also automatically find the correct conformation for this dataset (Fig. S5c)

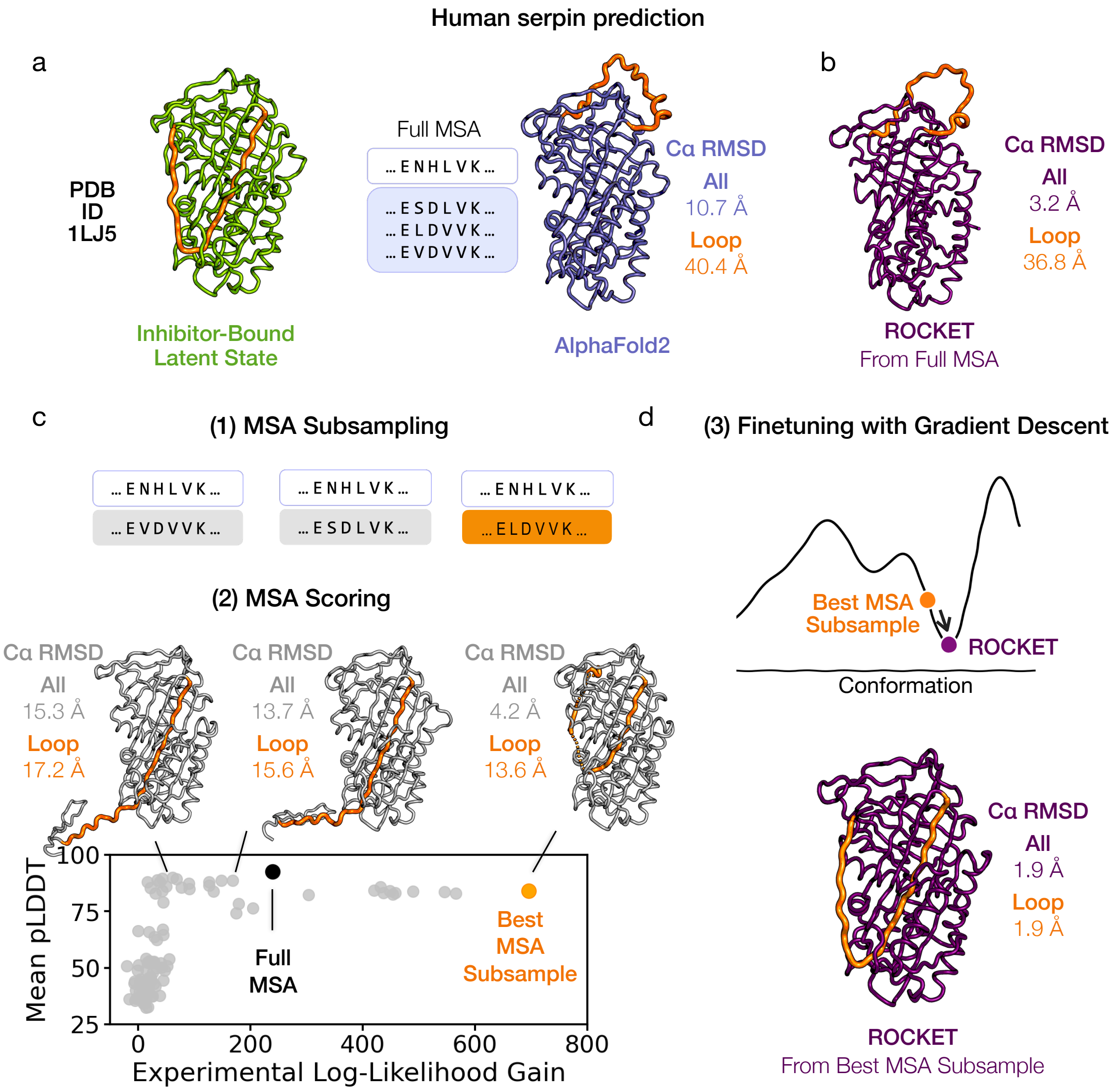

Fig. S7 Synergistic Integration of MSA Subsampling with Gradient-Based Optimization. Gradient-based refinement faces a limit when initial predictions deviate substantially from the target conformation. (a) The structure of a human serpin, plasminogen activator inhibitor-1 (PAI-1), in its latent state (PDB ID 1LJ5, 1.8 Å) illustrates this issue (left). A conventional AF2 prediction, generated from the full MSA, yields the metastable active conformation that diverges from the latent state captured by the experimental structure (right). (b) The latent state conformation is not accessible by gradient descent alone when ROCKET refinement is started from the full MSA (c) Subsampled MSAs (1) result in predictions that more closely resemble the experimental conformation (2). Ranking these predictions (bottom panel) by their experimental likelihoods identifies a better starting model for gradient-based refinement (samples are ordered along the x-axis by increasing gains in experimental likelihood), unlike pLDDT-based scoring, which does not (highest samples along the y-axis do not resemble the experimental conformation). (d) Gradient-based refinement of the best starting prediction from (c) results in a structure that closely resembles the latent conformation.

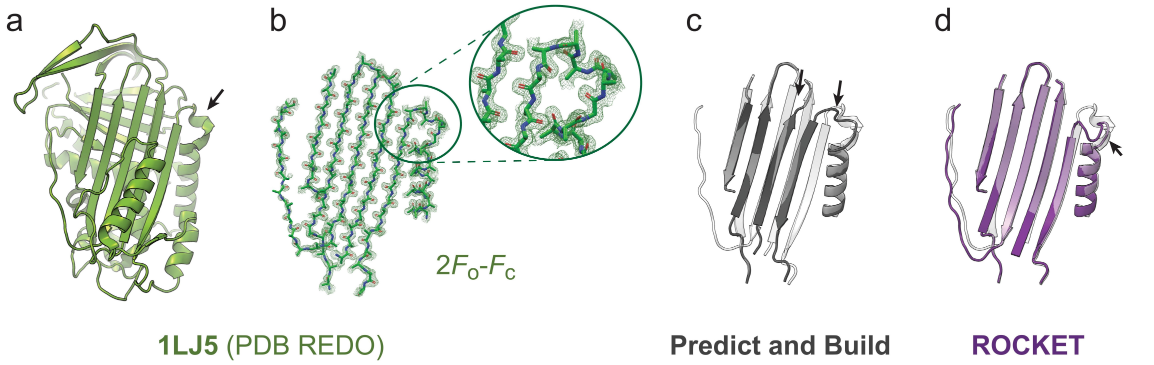

Fig. S8 ROCKET's internal optimization is robust to an unusual turn where PredictAndBuild is not. (a) Latent-state model of PAI-1 (PDB ID 1LJ5), also shown in Fig. S7. The arrow highlights the 85-91 loop containing unusual secondary structure. Both PredictAndBuild and ROCKET incorrectly reconstruct this loop despite excellent electron density. (b) 2Fo-Fc map (PDB-REDO) shown carved within 1.5 Å of backbone and Cβ atoms). (c) As an apparent consequence of this unusual turn, PredictAndBuild (dark gray) incorrectly models the topology of the central beta sheet, even when given the best subsampled MSA: two strands are modeled backwards and one strand is not modeled at all. The deposited structure is shown in outline. (d) ROCKET (purple), despite not correctly modeling the loop, models the beta sheet correctly.

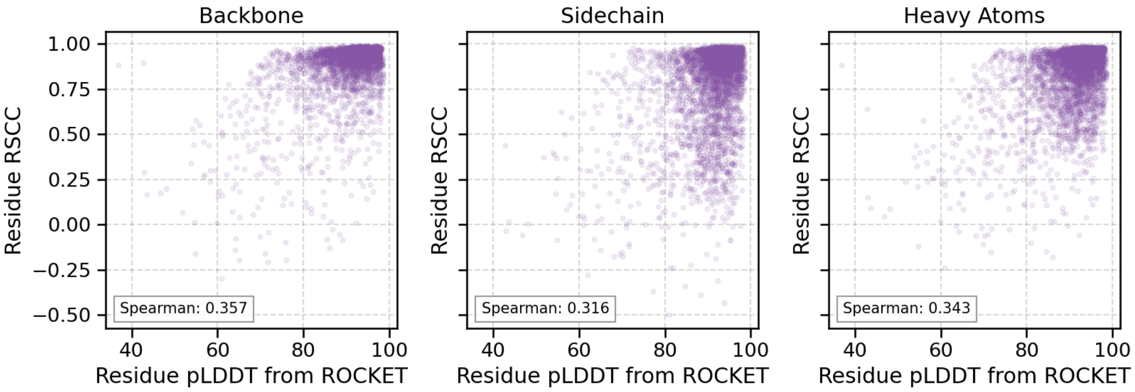

Fig. S9 Relationship between ROCKET pLDDT and Fit to Experimental Density. Residue pLDDT at the end of ROCKET refinement for all 27 high-resolution test cases are plotted against real-space correlation coefficients (RSCC) between the map calculated from the ROCKET model and the PDB-REDO map. Results are broken down by different atom types.

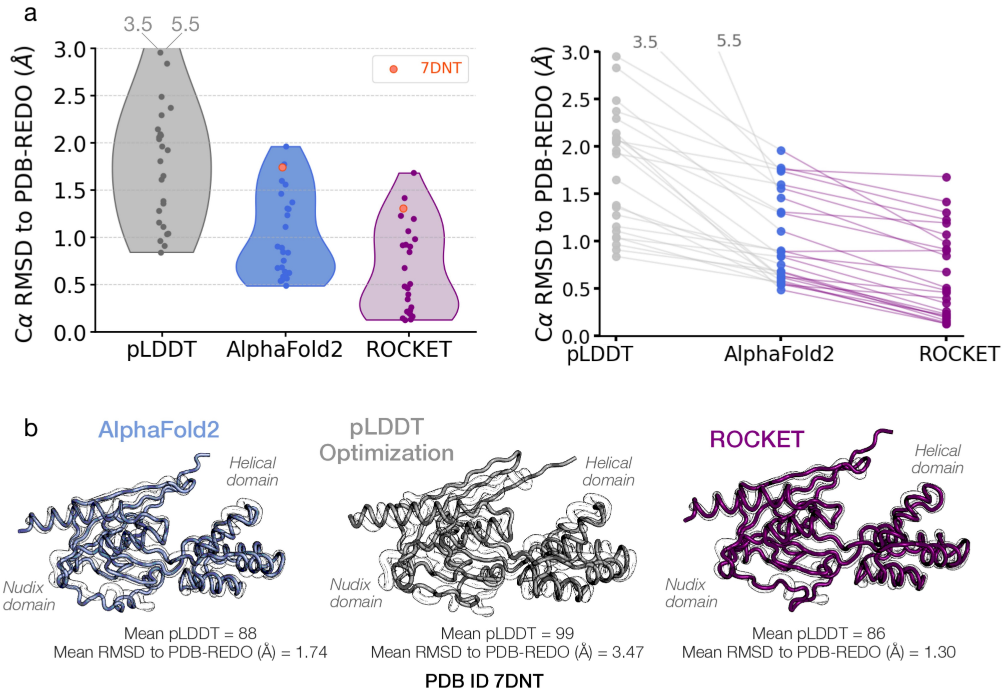

Fig. S10 Comparison with Confidence Maximization. To assess the added value of experimental data integration, we compare ROCKET's inference-time optimization to the results that can be obtained by maximizing AF2 model confidence. AF2 model confidence maximization has previously been used to explore alternate conformations for a given sequence, especially in the context of protein design. Depending on the task, different studies have optimized AF2's local confidence metric (predicted local-distance difference test – pLDDT) [4, 5, 10], its confidence of relative positioning between residues (predicted aligned error matrix – PAE matrix) [5], or interface confidence for complexes [2, 4]. We conducted a search in the MSA cluster profile space that maximizes pLDDT and found that AF2 does not easily produce experimentally observed conformations without further experimental information. (a) Cα RMSD values between PDB-REDO models and structures from standard AF2 inference, pLDDT maximization, and ROCKET's data-likelihood maximization. ROCKET consistently improves the match between AF2 predictions and experimental structures, while pLDDT maximization alone does not achieve comparable accuracy. (b) Example of the viral mRNA-decapping enzyme g5rp (PDB ID 7DNT) illustrating ROCKET's refinement capability. The initial AF2 prediction shows domain misalignment and secondary structure inaccuracies compared to the experimentally resolved conformation. ROCKET refines these discrepancies, while pLDDT maximization does not converge to the correct structure.

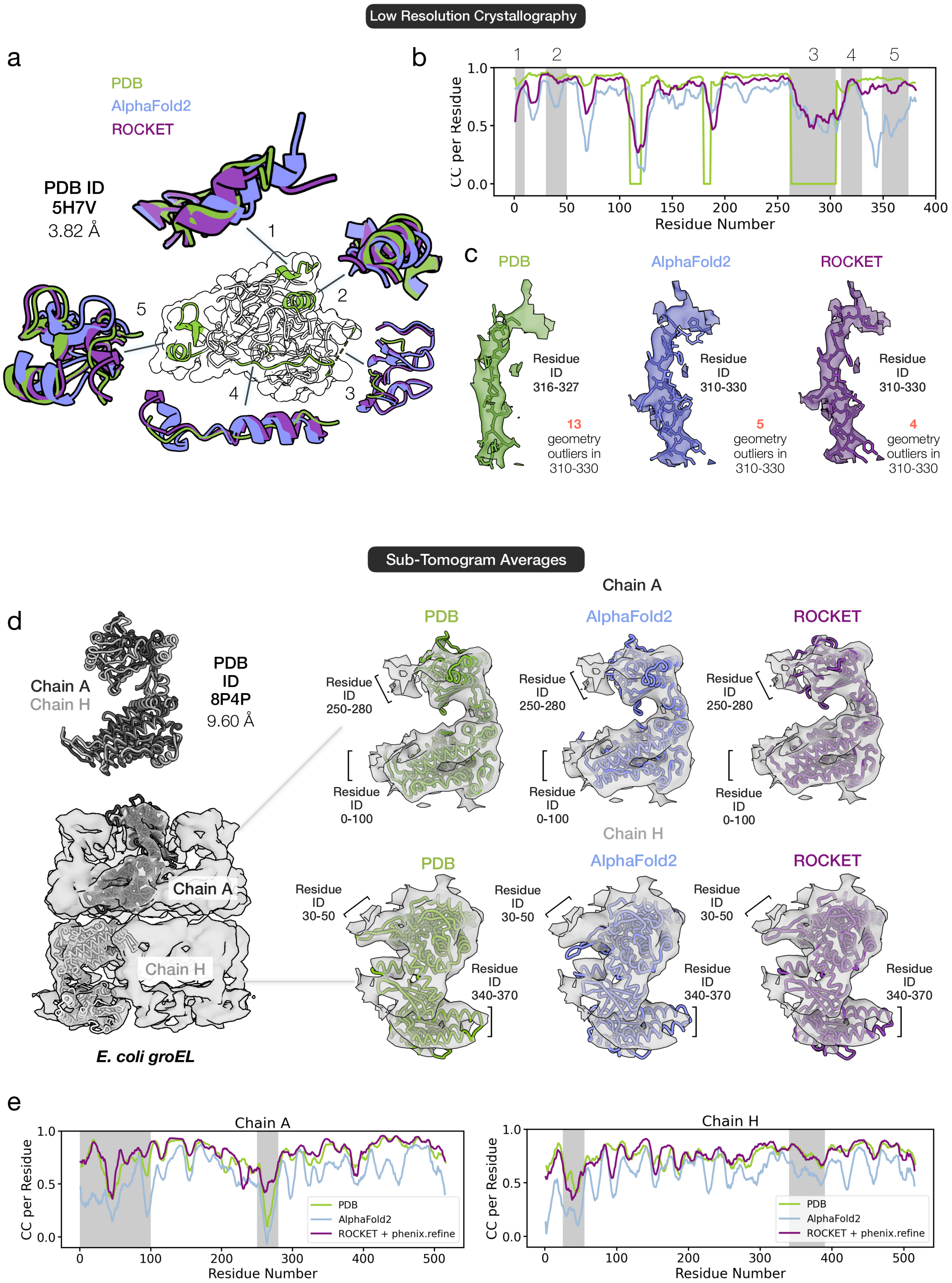

Fig. S11 Extracting Information from Low-Resolution Data. (a-c) Refinement of a 3.82 Å crystallographic dataset of the human protease inhibitor HAI-1 (PDB ID 5H7V). ROCKET accurately recovers backbone structures in regions where the data support the deposited model (regions 1, 2, and 5) and preserves the AF2 prediction in poorly defined regions (region 3). In region 4, where manual model building is hindered by noisy density and seemingly incorrect sequence register in the deposited model, ROCKET improves the initial AF2 prediction without introducing new geometric outliers. (d-e) Refinement of a 9.60 Å sub-tomogram average of *E. coli* GroEL (PDB ID 8P4P). ROCKET successfully predicts two distinct subunit conformations observed in the GroEL heptameric rings. Maps computed using the refined models correlate with the experimental map at a level comparable to human-built models. For the chain A conformer, ROCKET explores a broader conformational space, with its final model achieving a higher average RSCC of 0.5 for residues 250–280 (marked by a star) than the deposited model's RSCC of 0.2.

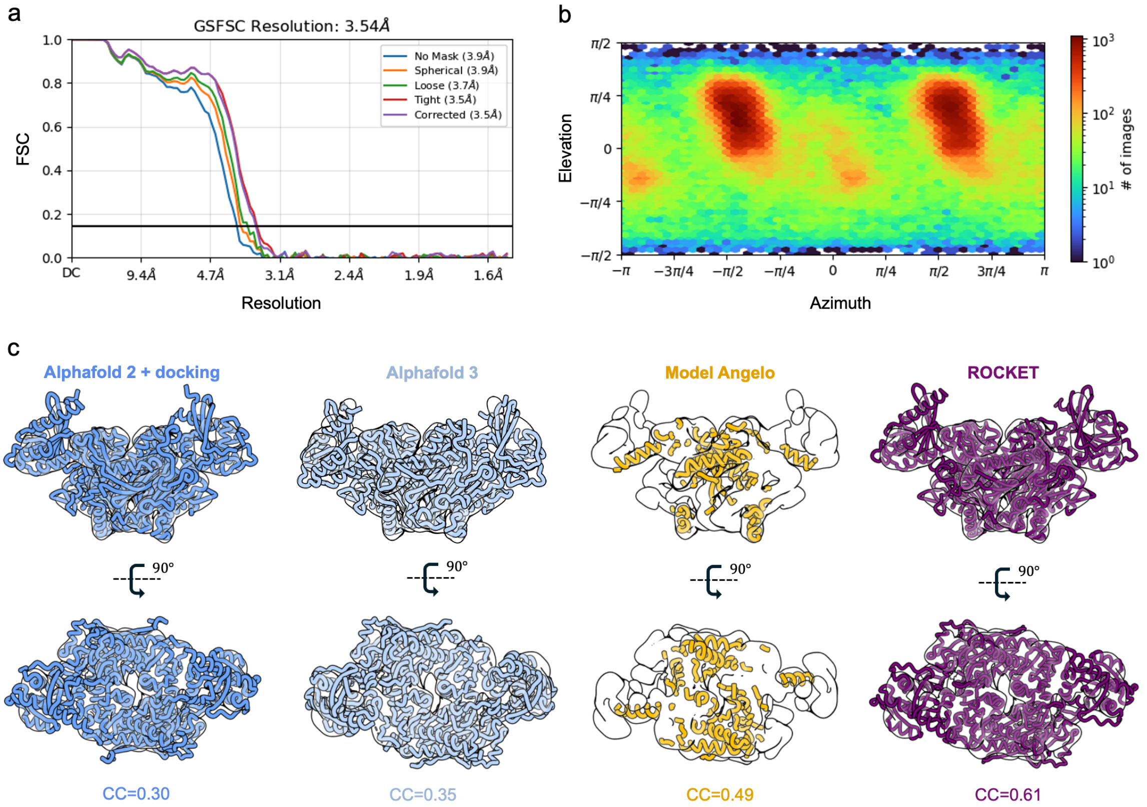

Fig. S12 ROCKET-based refinement on single-particle cryo-EM data with strong preferred orientation: the structure of a 2:2 assembly of human Ser/Thr phosphatase PPM1H and Rab8a based. (a) FSC plot from CryoSparc. (b) Single particle images orientation diagnosis from cryosparc, showing the preferred orienteintaion issue. (c) Comparison of model building methods for the 3.4 Å cryo-EM map of the PPM1H-Rab8a dimeric complex. A model generated by docking individually predicted AlphaFold2 chains (using emplace_local[8] tool in chimeraX) yielded a real-space correlation coefficient (CC) of 0.30. An slightly improved model (CC = 0.35) was obtained by applying symmetry operations to an AlphaFold3-predicted 1:1 heteromer; direct AlphaFold3 prediction of the 2:2 complex was unsuccessful. While the *de novo* tool ModelAngelo (v1.0.13 with default settings) improved the local model-to-map fit, its overall model completeness was low ($< 20\%$). In contrast, ROCKET generated a complete model with the highest overall CC.

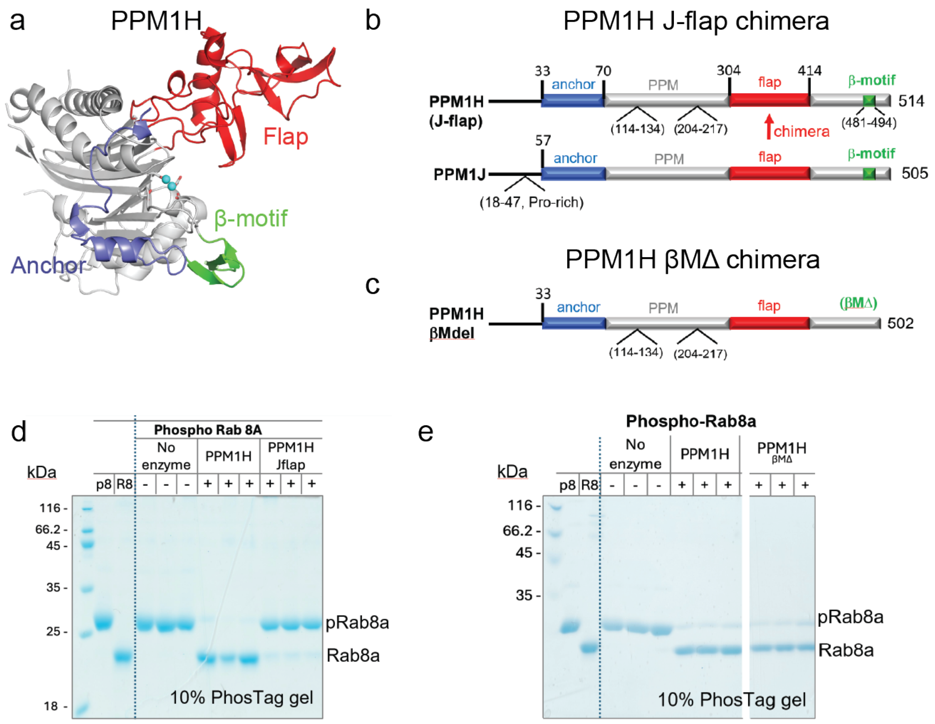

Fig. S13 Biochemical data support two key features of the ROCKET model of the PPM1H-Rab8a interaction. (a) Structure of PPM1H with Mg2+ ions (cyan spheres) at the active site (PDB ID 7L4J). PPM1H adopts a conserved PPM fold punctuated by an extended flap domain (110 residues) and a β-sheet motif (β-motif, 15 residues) adjacent to the active site. The flap domain and beta-motif are conserved in PPM1J and PPM1M. (b) construction of the "J-flap chimera": PPM1H Jmut containing the flap domain of PPM1J (80% sequence conservation). (c) construction of the β-motif knockout ($\beta M\Delta$). (d) Phosphatase activity assay for the J-flap chimera: the cognate PPM1H flap domain is essential for specific dephosphorylation of Rab8a. (e) Phosphatase activity assay for the beta-motif knockout. In contrast to the J-flap chimera, removal of the β-motif has no effect on catalysis consistent with its lack of direct interactions with Rab8a in the ROCKET model, whereas a recent AlphaFold3 model appears to place the β-motif closer to Rab8a [1]. 10% PhosTag gels with triplicate repeats. High-resolution versions are available in the accompanying Zenodo deposition (https://zenodo.org/uploads/15084558).

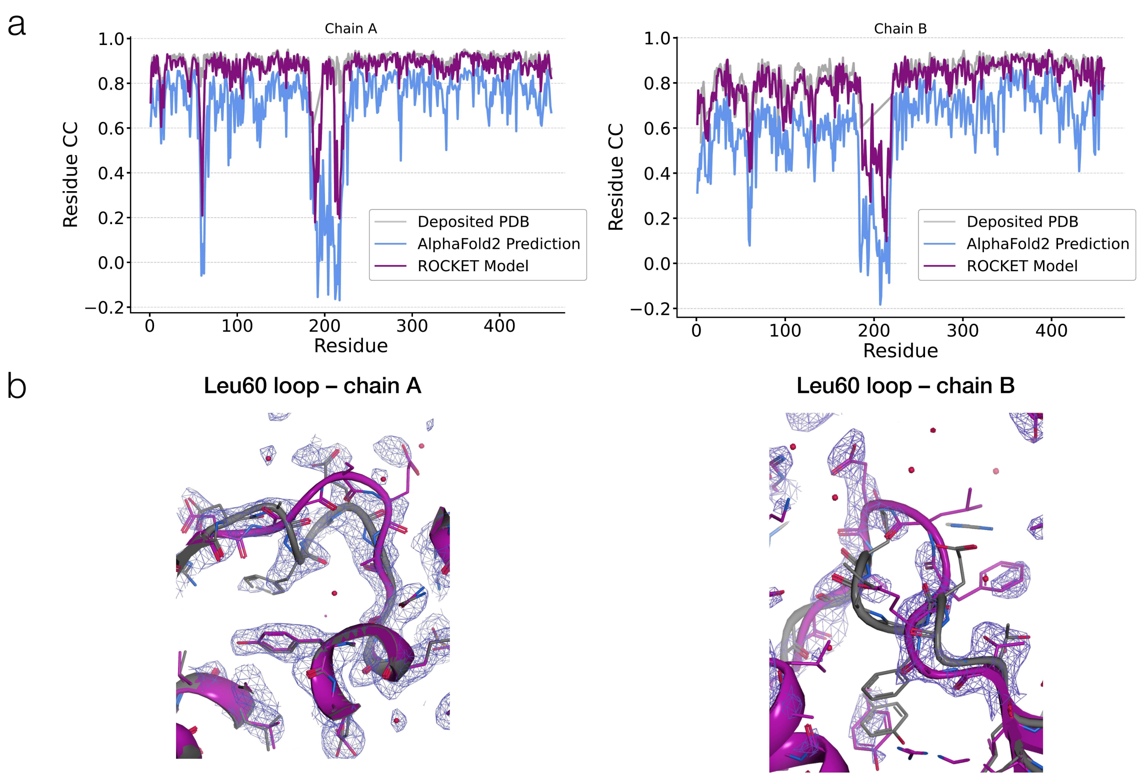

Fig. S14 Time-Resolved Serial Femtosecond Crystallography Dataset Rebuilding. ROCKET rebuilding of the time-resolved 10 μs intermediate of photolyase during DNA repair. Per-residue RSCC for relevant models and their respective map: AlphaFold2, PDB ID 8OAY deposition, and ROCKET. Because phases from the AF2 model are poorer, RSCC was computed using phases from the ROCKET model. For the highly disordered loop region (residues 186-200), which is not modeled in the deposited structure, ROCKET builds a possible model that is geometrically plausible and has positive correlation to the map. (b) In the region of Leu60, ROCKET samples a conformation that does not fit the data as well as the deposited structure for chain A, but, for chain B, represents an interesting alternative model from the deposited one. Maps shown at +1.3 RMS.)

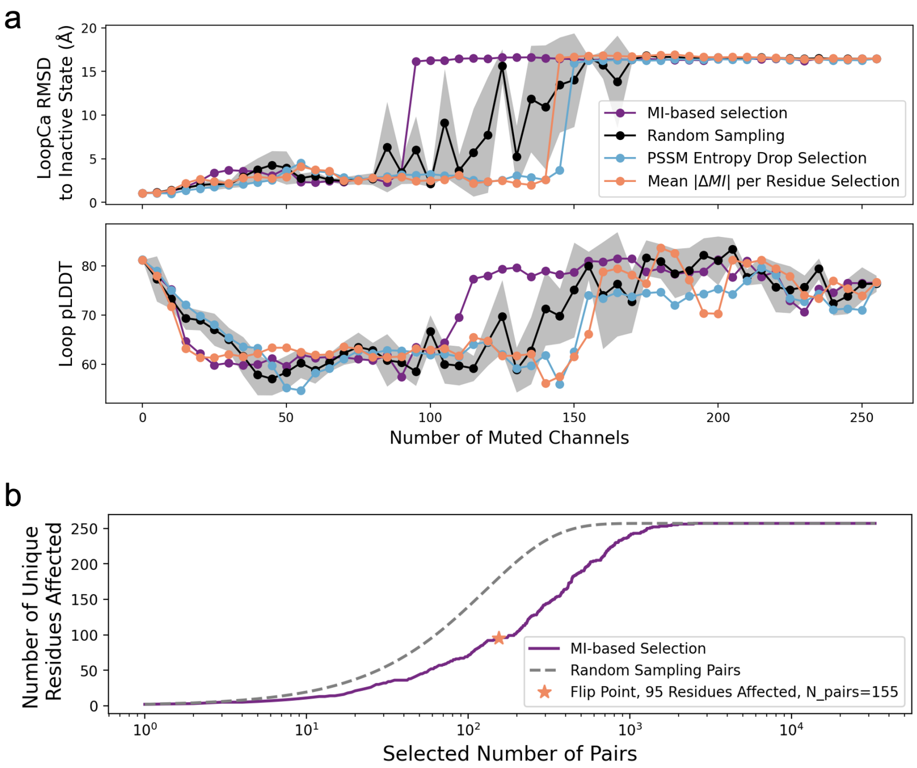

Fig. S15 Mutual Information Highlights Distributed Residue Signals Driving Conformational Transitions (a) Causal testing via profile bias muting. Top: Activation Loop Ca RMSD to the inactive state vs. number of muted channels. Muting channels based on MI difference (purple) reverts the structure to its active state more effectively than other methods including random muting (gray; mean and standard deviation over 5 independent trials per point), PSSM entropy drop selection (light blue) and average absolute MI change selection (light orange). For PSSM entropy drop selection, the profile matrix is averaged along the cluster axis to form a PSSM-like matrix, the entropy change for each residue before and after ROCKET refinement is computed, and residues are picked from highest to lowest entropy drop. For average absolute MI change selection, the mean $|\Delta MI|$ per residue is calculated and residues are selected from highest to lowest. Bottom: Corresponding pLDDT of the Activation Loop. (b). The cumulative number of unique residues affected by the top-ranked $|\Delta MI|$ pairs (purple) compared to the expected number from random sampling (dashed gray). The top 155 pairs in the $|\Delta MI|$ ranking involve only 95 unique residues (star) to flip the state, substantially fewer than the number of residues affected by 155 randomly selected pairs. The expectation for random sampling is calculated analytically with $N(1 - \mathrm{C}(\mathrm{C}(N-1, 2), K)/\mathrm{C}(\mathrm{C}(N, 2), K))$, where N is the number of residues, K is the number of sampled pairs, C is the combination operator.

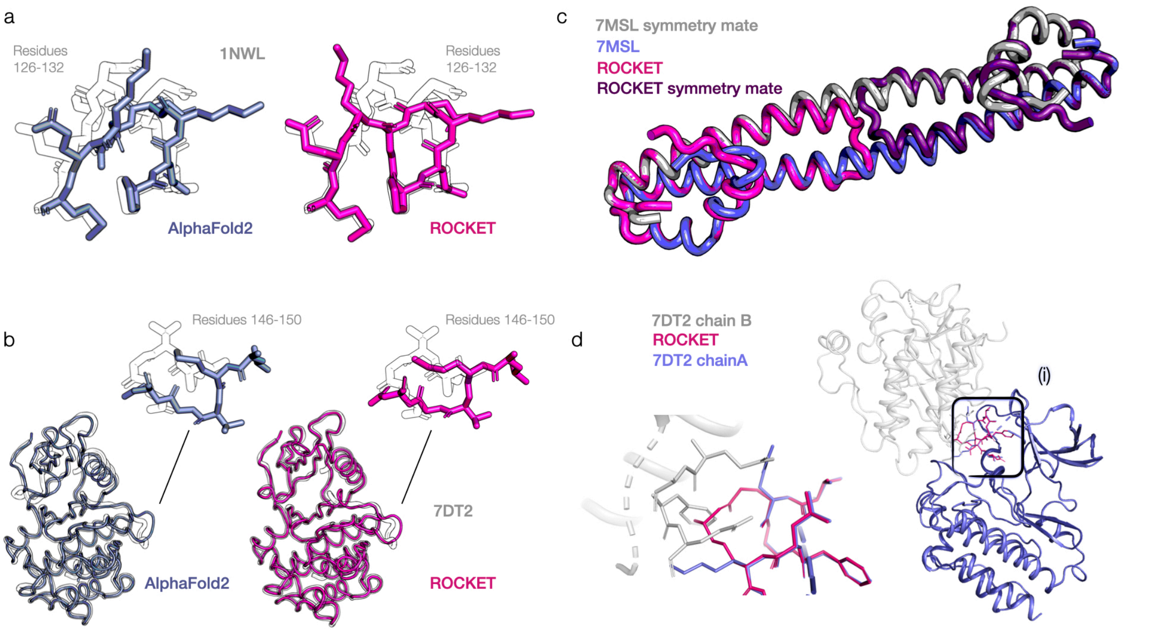

Fig. S16 Current Shortcomings in ROCKET Model Building. (a-b) We notice that ROCKET can fail to flip small loops (3-4 residues in length) that contain long sidechain residues. (c-d) Due to OpenFold's lack of awareness of crystal contacts, ROCKET may struggle to converge to certain lattice-dependent conformations (PDB ID 7SML, a dimer with crystallographic symmetry), or to account for the presence of another chain in the asymmetric unit (PDB ID 7DT2).

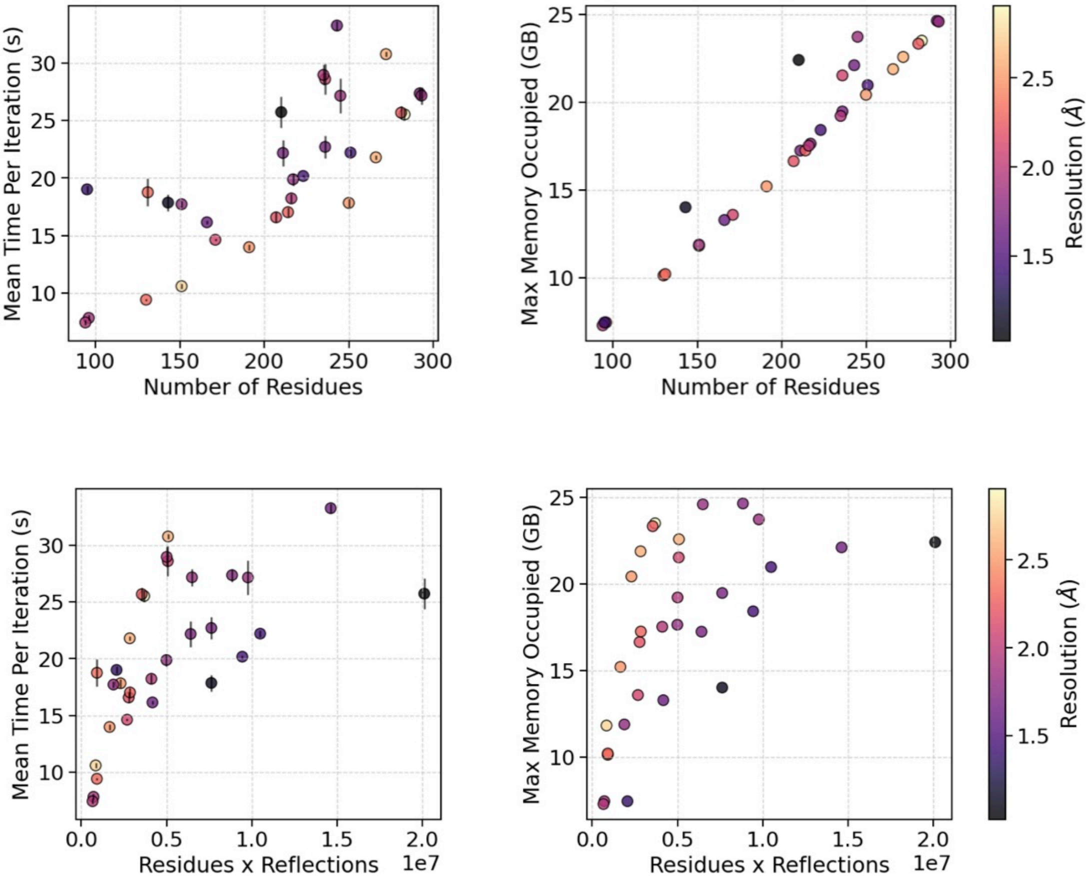

Fig. S17 Memory and Computation Time Requirements for ROCKET on a Nvidia 40 GB A100 GPU. Here, we report the mean time per iteration and the peak GPU memory occupancy for 31 systems, plotted against the number of residues (protein size) and against the product of number of residues and number of reflections (a proxy for resolution). In the time plot, each value is the mean per-iteration running time over 100 ROCKET iterations on the same system, and the error bar shows the standard deviation across those 100 iterations. For the largest system in our high resolution benchmark set, 7FIU (293 residues, 1.8 Å resolution), Phase 1 (100 steps) required 26 min 14 s, while Phase 2 converged after ≈200 steps, requiring an additional 1 hr 11 min 03 s. For the smallest system, 7DMS (94 residues, 1.96 Å resolution), Phase 1 (100 steps) required 7 min 02 s, and Phase 2 (500 steps) required 34 min 43 s.

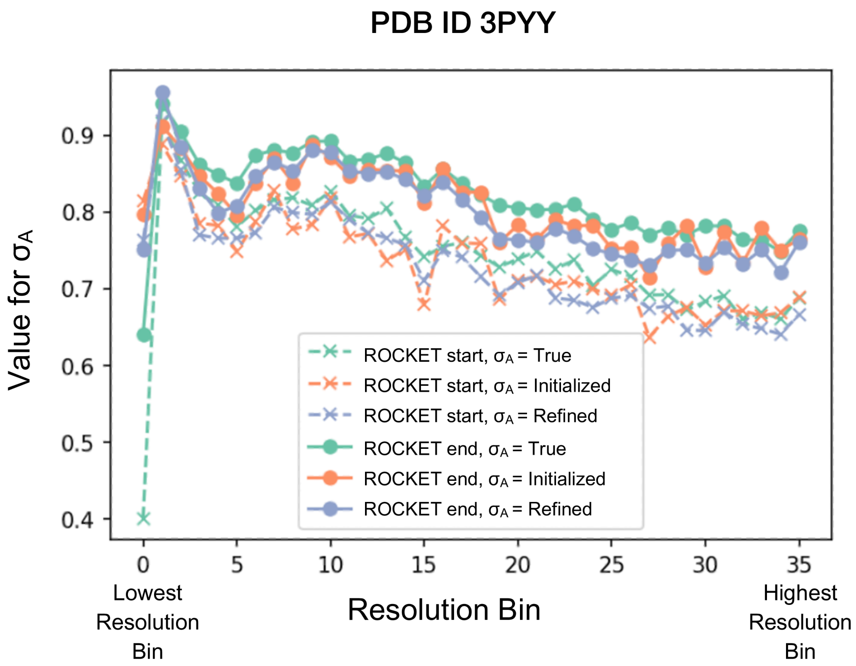

Fig. S18 Refinement of Crystallographic σ_A with the Working Set of Reflections. We find that refining σ_A values with ROCKET using the working set of reflections does not lead to meaningful overfitting. Here we plot σ_A values for each resolution bin for the starting (ROCKET start) and final (ROCKET end) iterations of ROCKET refinement for the PDB ID 3PYY dataset of the c-Abl kinase. In orange, we show the initialized values for the iteration and, in blue, the values after the σ_A refinement described in Methods. For comparison, we also plot, in green, the σ_A values that can be computed using the PDB REDO model phases and that we use as the basis for "true" σ_A comparison.

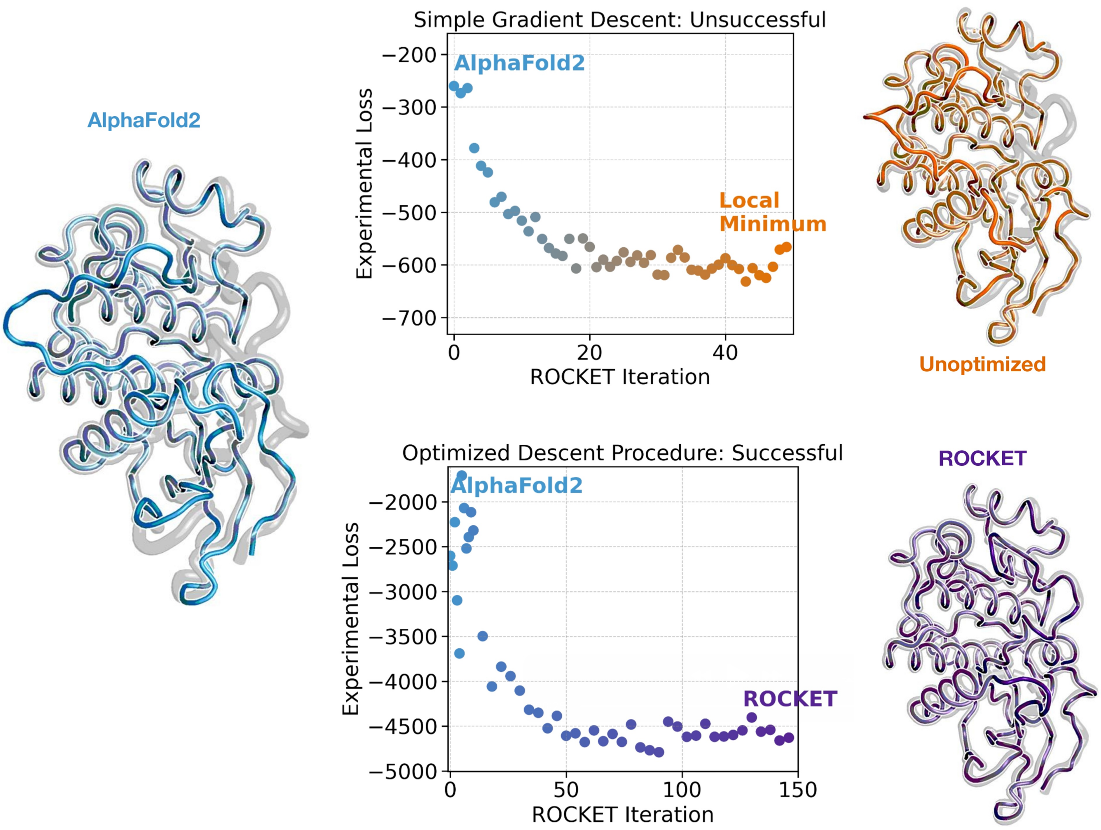

Fig. S19 Optimization of ROCKET's Gradient Descent Procedure. Starting from the initial AlphaFold2 prediction for c-Abl kinase, two ROCKET runs are shown for the refinement to a drug-bound crystallographic dataset, where the deposited experimental conformation is shown in gray (PDB ID 3PYY). The top panel displays the refinement results when ROCKET is run with its phase 2 parameters (a low learning rate and an unoptimized gradient descent procedure). The experimental loss (negative LLG score) decreases over the first iterations but plateaus at a local minimum (orange structure), where the main activation loop has not reached the experimental conformation. The bottom panel displays the refinement results when running ROCKET's phase 1, outlined in the Methods. Through this optimized procedure, ROCKET (purple structure) can refine the full backbone so that it matches the experimental conformation.

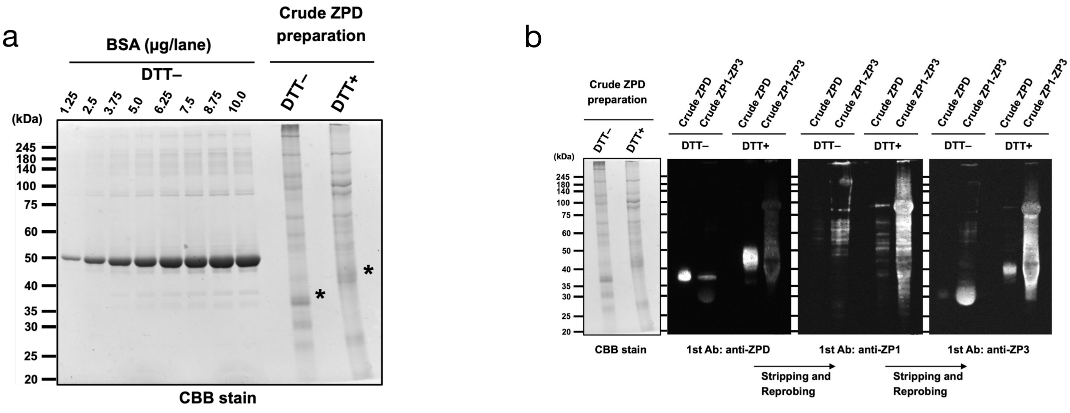

Fig. S20 ZPD Sample Preparation. (a) 2.4 μL crude ZPD preparation and 10 μL BSA solutions at varying concentrations were separated by SDS-PAGE and stained with CBB. ZPD bands are indicated by asterisks, based on Western blotting with anti-ZPD antiserum (panel b). DTT+: reducing conditions; DTT–: non-reducing conditions. (b) Crude ZPD preparation and/or crude ZP1–ZP3 complex were separated by SDS-PAGE and analyzed by CBB staining (identical to the right side of panel a) or Western blotting with anti-ZPD, anti-ZP1, or anti-ZP3 antisera (left to right). The same membrane was sequentially stripped and reprobed with these antisera [6]. ZPD was confirmed to be highly enriched in the crude ZPD preparation.

Supplementary Tables

Code	n_res	TM_score	Spacegroup	Resolution (Å)	RMSD(AF2, deposited) (Å)
7TFQ	292	1.000	P 21 21 2	1.75	0.48
7RM7	228	0.938	P 21 21 21	1.03	0.54
7U2R	245	0.999	P 61 2 2	1.85	0.54
7DMS	94	0.424	P 21 21 21	1.96	0.56
7UNN	260	0.902	P 21 21 21	1.45	0.58
7TBS	238	0.945	I 2 2 2	1.96	0.62
7KZH	202	0.979	P 3 2 1	2.49	0.62
7EYJ	95	0.999	P 62	1.38	0.64
7T26	144	0.999	P 41 21 2	1.14	0.67
7T7Y	154	0.990	P 2 21 21	1.81	0.68
7VNX	223	0.996	P 61	1.80	0.75
7RPY	256	0.957	P 32 2 1	1.67	0.84
7QDV	100	0.994	P 43 21 2	1.90	0.84
7RAW	251	0.975	I 21 3	2.10	0.89
7O51	207	0.994	P 41 21 2	2.20	0.90
7EJG	237	0.998	P 61	1.68	1.11
7ECD	272	0.997	I 41	2.60	1.31
7EDC	249	0.989	C 1 2 1	1.95	1.46
7S3L	269	0.997	P 43 21 2	2.60	1.56
7AOJ	179	0.910	P 61	1.63	1.60
7DNT	250	0.990	P 21 21 2	2.50	1.74
7FIU	293	0.943	P 21 21 21	1.84	1.77
7SEZ	221	0.990	C 2 2 21	1.70	1.75
7TRW	217	0.994	P 62	2.28	1.96

Table S1 Table of high-resolution crystallographic benchmark dataset used for ROCKET. n_res indicates the number of residues.

Table S2 Data collection parameters for ZPD low resolution dataset

Parameter	Value
Magnification	165,000×
Voltage (kV)	300
Electron exposure ($e^-/Å^2$)	50
Defocus range (μm)	-0.7 – -2.8 (0.3 steps)
Pixel size (Å)	0.7336
Movies (no.)	11,596
Initial particles (no.)	1,113,548
Final particles (no.)	317,745
Symmetry imposed	Helical (rise 71.4 Å, twist 120.8°)
Nominal map resolution (Å)	8.6
Gold standard (cryoSPARC) FSC threshold	0.143

Table S3 Data collection parameters for ZPD medium resolution dataset

Parameter	Value
Magnification	165,000×
Voltage (kV)	300
Electron exposure ($e^-/Å^2$)	52 (Data 1); 53.06 (Data 2)
Defocus range (μm)	-0.7 – -2.8 (0.3 steps)
Pixel size (Å)	0.7336
Movies (no.)	7,389 (Data 1); 12,564 (Data 2)
Initial particles (no.)	2,031,564
Final particles (no.)	498,339
Symmetry imposed	C1
Nominal map resolution (Å)	4.6
Gold standard (cryoSPARC) FSC threshold	0.143

Supplementary References

[1] Ayan Adhikari, Aashutosh Tripathi, Claire Y Chiang, Pemba Sherpa, and Suzanne R Pfeffer. Allosteric regulation of the Golgi-localized PPM1H phosphatase by Rab GTPases modulates LRRK2 substrate dephosphorylation in Parkinson's disease. *Journal of Biological Chemistry*, page 110679 (2025).

[2] Patrick Bryant and Frank Noé. Improved protein complex prediction with AlphaFold-multimer by denoising the MSA profile. *PLOS Computational Biology*, 20(7):e1012253 (2024).

[3] Clark C Fjeld and John M Denu. Kinetic analysis of human serine/threonine protein phosphatase 2cα. *Journal of Biological Chemistry*, 274(29):20336–20343 (1999).

[4] Christopher Frank, Ali Khoshouei, Lara Fuß, Dominik Schiwietz, Dominik Putz, Lara Weber, Zhixuan Zhao, Motoyuki Hattori, Shihao Feng, Yosta de Stigter, Sergey Ovchinnikov, and Hendrik Dietz. Scalable protein design using optimization in a relaxed sequence space. *Science*, 386(6720):439–445 (2024).

[5] Michael Jendrusch, Jan O. Korbel, and S. Kashif Sadiq. AlphaDesign: A de novo protein design framework based on AlphaFold. *BioRxiv*, page 10.1101/2021.10.11.463937 (2021).

[6] Hiroki Okumura, Takahiro Sato, Rio Sakuma, Hideaki Fukushima, Tsukasa Matsuda, and Minoru Ujita. Identification of distinctive interdomain interactions among zp-n, zp-c and other domains of zona pellucida glycoproteins underlying association of chicken egg-coat matrix. *FEBS Open Bio*, 5:454–465 (2015).

[7] Ali Punjani, John L. Rubinstein, David J. Fleet, and Marcus A. Brubaker. cryoSPARC: algorithms for rapid unsupervised cryo-EM structure determination. *Nature Methods*, 14(3):290–296 (2017).

[8] Randy J Read, Claudia Millán, Airlie J McCoy, and Thomas C Terwilliger. Likelihood-based signal and noise analysis for docking of models into cryo-em maps. *Biological Crystallography*, 79(4):271–280 (2023).

[9] Dieter Waschbüsch, Kerryn Berndsen, Pawel Lis, Axel Knebel, Yuko PY Lam, Dario R Alessi, and Amir R Khan. Structural basis for the specificity of PPM1H phosphatase for Rab GTPases. *EMBO reports*, 22(11):e52675 (2021).

[10] B. I. M. Wicky, L. F. Milles, A. Courbet, R. J. Ragotte, J. Dauparas, E. Kinfu, S. Tipps, R. D. Kibler, M. Baek, F. DiMaio, X. Li, L. Carter, A. Kang, H. Nguyen, A. K. Bera, and D. Baker. Hallucinating symmetric protein assemblies. *Science*, 378(6615):56–61 (2022).

[11] Christopher J. Williams, Jeffrey J. Headd, Nigel W. Moriarty, Michael G. Prisant, Lizbeth L. Videau, Lindsay N. Deis, Vishal Verma, Daniel A. Keedy, Bradley J. Hintze, Vincent B. Chen, Swati Jain, Steven M. Lewis, W. Bryan Arendall III, Jack Snoeyink, Paul D. Adams, Simon C. Lovell, Jane S. Richardson, and David C. Richardson. MolProbity: More and better reference data for improved all-atom structure validation. *Protein Science*, 27(1):293–315 (2018).
